# Dark-centred umbels in Apiaceae: diversity, development and evolution

**DOI:** 10.1093/aobpla/plad065

**Published:** 2023-09-13

**Authors:** Regine Claßen-Bockhoff, Ferhat Celep, Yousef Ajani, Lisa Frenken, Kerstin Reuther, Musa Doğan

**Affiliations:** Institute of Organismic and Molecular Evolution (IOME), Department of Biology, Johannes Gutenberg-University, Saarstraße 21, 55099 Mainz, Germany; Faculty of Science and Letters, Department of Biology, Kırıkkale University, Kırıkkale, Yahşihan, P.O. 71450, Turkey; Institute of Organismic and Molecular Evolution (IOME), Department of Biology, Johannes Gutenberg-University, Saarstraße 21, 55099 Mainz, Germany; Department of Botany, Research Institute of Forests and Rangelands, P.O. Box 13185-116, Tehran, Iran; Institute of Organismic and Molecular Evolution (IOME), Department of Biology, Johannes Gutenberg-University, Saarstraße 21, 55099 Mainz, Germany; Institute of Organismic and Molecular Evolution (IOME), Department of Biology, Johannes Gutenberg-University, Saarstraße 21, 55099 Mainz, Germany; Department of Biological Sciences, Middle East Technical University Ankara, Çankaya, P.O. 06800, Turkey

**Keywords:** Anatolia, *Artedia squamata*, beetle marks, dark-centred umbels, *Daucus* alliance, *Echinophora trichophylla*, floral unit meristem, generalized pollination, homology, multicyclic protandry, mutation, umbel development

## Abstract

The wild carrot (*Daucus carota*) is famous for its dark flowers in the umbel centre. Several studies have been conducted to figure out their functional significance, but the evolution of the dark centre remains an enigma. In the present paper, we consider all known apioid species with dark-centred umbels to get a deeper understanding of their biology and evolution. Based on herbaria studies, literature and field work, we reconstructed the distribution area of 10 species (7 genera, 6 clades) of Apiaceae-Apioideae. To recognize homology of the dark structures, developmental studies were conducted in *Artedia squamata* and *Echiophora trichophylla* Field studies included architecture, flower morph distribution (andromonoecy) and flowering sequence within the plants, abundancy and behaviour of umbel visitors and preliminary manipulation experiments (removal/adding of dark structures). The dark structures are not homologous to each other. In the *Daucus* alliance, central flowers or umbellets are conspicuous, whereas in other species dark brush-like (*A. squamata*) or club-shaped structures (*Dicyclophora persica*, *Echinophora trichophylla*, *Tordylium aegyptiacum*, *T. cappadocicum*) develop from a naked receptacle. Species are andromonoecious, have a modular architecture and flower in multicyclic protandrous sequence. Among the many umbel visitors, beetles were the most abundant group. Only visitors found on umbels in both flowering phases were recognized as possible pollinators. Manipulation experiments indicated that the dark structures influence the behaviour of some, but not all umbel visitors. In *Echinophora trichophylla*, a massive gall infection was observed. It is evident that the dark structures evolved several times in parallel. The brush- and club-shaped structures are interpreted as the results of mutations affecting umbel development. Dark umbel centres are most likely stabilized by selection due to their general adaptive function. Their appearance in an area known as a hotspot of beetle pollination gives rise to the assumption that they may act as beetle marks.

## Teaser

The wild carrot is famous for its red flower in the umbel centre. The function of this flower has been discussed since the time of Darwin. Experiments have led to the general conclusion that it is adaptive addressing at least part of the unspecialized visitors and pollinators. We show that *Daucus carota* is not the only species with dark umbel-centres. At least 10 apioid species from 7 genera and 6 clades have conspicuous dark structures in their umbels. It is evident that the morphologically diverse structures evolved in parallel. Some receptacle structures appear to be inherited mutations linked with the peculiarities of the umbel meristem. As the diversity centre of the species with dark-centred umbels is SW Asia, an area known as a hotspot of beetle pollination, we speculate that the dark umbel centres may act as beetle marks not only but primarily attracting beetles as pollinators.

## INTRODUCTION

**Figure 1.**
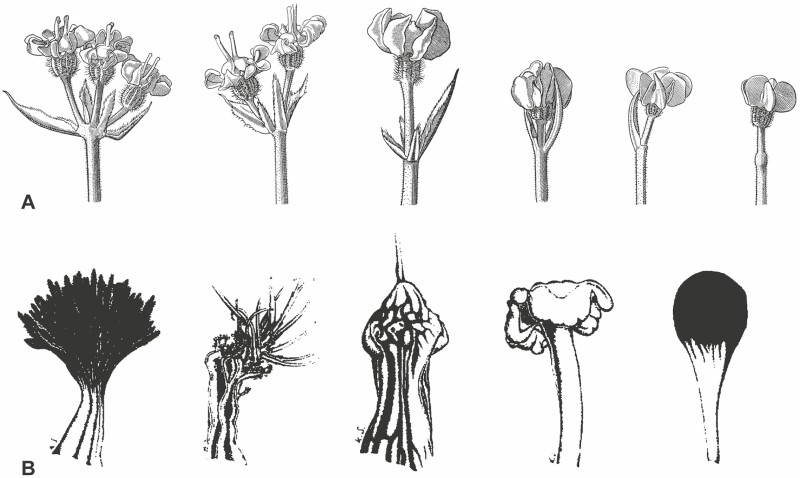
Dark structures in apioid umbels. (A) *Daucus carota* subsp. *carota*. Diversity of dark flowers in the umbel centre. Homology with a terminal umbellet is corroborated by the presence of involucellar bracts. Taken from Troll (1957). (B) Massive structures in the umbel centre of *Artedia squamata*, *Exoacantha heterophylla*, *Echinophora trichophylla* and *Tordylium aegyptiacum*. *Dicyclophlora persica* (from left to right). Taken from Schmidt and Magin (1997).

Apiaceae, particularly the largest subfamily Apioideae, is easily recognizable by white or yellow umbels consisting of umbellets. Though looking rather uniform, these umbels are surprisingly diverse in size, density, flower dimorphism and bract formation (Reuther and Claßen-Bockhoff 2010). One of the most remarkable characters is the appearance of one or few dark flowers in the umbel centre of the wild carrot *Daucus carota* (Fig. 1A).

The dark flower already raised the interest of Darwin (1877) who assumed that it had no function but rather represented a remnant from a former evolutionary time. After him, the dark centre has been the matter of several studies and interpreted as a structure mimicking an insect and/or gall, protecting plants from grazing animals or just being an inherited ‘abnormality’ (summarized by Schmidt and Magin 1997; Lamborn and Ollerton 2000; Goulson *et al.* 2009).

Almost all field studies have been conducted with *D. carota* subsp. *carota* in Europe (Daumann 1973; Lamborn and Ollerton 2000; Goulson *et al*. 2009), the USA (Westmoreland and Muntan 1996) or W Turkey (Gonzalez *et al*. 2018). Pérez-Bañón *et al.* (2007) observed insect visitors in *D. carota* subsp. *commutatus* on a small Spanish island and Eisikowitch (1980) in *D. carota* subsp. *maximus* Ball in Israel. The latter also conducted choice experiments with naïve house flies (*Musa domestica*) in the lab testing the attraction of the black spots in *D. carota* subsp. *maximus* and *Artedia squamata*. He found that the flies preferred the black-spotted umbels (and corresponding dummies) and hypothesized that the black spots served to stimulate aggregative behaviour of flies and, thus, increased the number of visitors. Westmoreland and Muntan (1996); Lamborn and Ollerton (2000) and Goulson *et al*. (2009) tested this *flycatcher effect* by manipulation experiments. However, their results were rather controversial and led to the general conclusion that the black spots might be adaptive for some insects whereas others were not affected. Altogether, the evolutionary significance of the dark flower in *Daucus* ‘remained an enigma’ (Lamborn and Ollerton 2000).

*Daucus carota, D. carota* subsp. *maximus* and *Artedia squamata* are not the only apioid species with dark-centred umbels. Schmidt and Magin (1997) listed nine species (and subspecies) from seven genera presenting either dark flowers (*D. carota* subsp*. carota*, *D. carota* subsp*. maximus*, *D. guttatus*, *D. broteri*) or brush- (*Artedia squamata*) or club-shaped structures (*Dicyclophora persica*, *Echinophora trichophylla*, *Exoacantha heterophylla*, *Tordylium aeyptiacum*) of unknown homology (Fig. 1B). Interestingly, except *Daucus carota* with an almost world-wide distribution, all these species are distributed from the E Mediterranean area to SW- and C-Asia raising the question what might be special in this area to evolve and maintain black spots. Except *D. carota* (e.g. Koul *et al*. 1989; Grzebelus *et al.* 2011, and aforementioned references), little is known about the biology of these species. It is assumed that they share the basic characters of Apiaceae like self-compatibility (e.g. Webb 1981; Koul *et al.* 1984; Spalik 1991; Jury 1996; Schlessman 2010) and promiscuous pollination (e.g. Bell 1971; Lindsey 1984; Lindsey and Bell 1985; Koul *et al*. 1993; Ajani and Claßen-Bockhoff 2021). Most species have a modular architecture with an ordinal flowering sequence, that is, the main umbel flowers first followed by all umbels of the first branch order, then those of the second order and so on. Species are usually andromonoecious, producing perfect and staminate flowers on the same individual, and protandrous, presenting pollen before the stigmatic surface becomes receptive.

The present paper gives a survey about the diversity, distribution area and phylogenetic position of apioid species with dark-centred umbels. It includes developmental studies elucidating the morphological nature of the dark structures and field studies on natural populations of *Artedia squamata *and *Echinophora trichophylla*. Linking morphology and pollination with reproductive biology promises a deep insight into the evolution of dark-centred umbels.

## MATERIAL AND METHODS

### Search for species and localities

To identify apioid species with dark-centred umbels, we checked the herbaria of Edinburgh (RBGE), Tehran (TARI) and Ankara (GAZI), as well as the relevant floras of Europe (Tutin *et al.* 1968), Turkey (Davis 1972), Iran (Rechinger and Hedge 1987), Cyprus (Meikle 1977) and Palestina (Post 1932). Surprisingly, though the dark umbel centres are usually very conspicuous, they are only rarely mentioned in the taxonomic literature. To get a deeper understanding of the diversity and evolutionary significance of dark-centred umbels, we started field investigations in Anatolia (the Asian part of Turkey) aiming to find natural populations of as many dark-centred apioid species as possible. Extended field trips in Turkey and Iran aimed to verify the information about localities and even find new species with dark-centred umbels.

At each locality, we collected some basic data including altitude and vegetation type. For each population, we roughly recorded the approximate number and density of individuals and the range of insect visitors. We made some notes of characteristic features of the respective species, particularly of the umbel construction, general plant architecture and flowering sequence.

### Case studies

In most species, we collected preliminary data serving as a starting point for future studies. However, in *Daucus carota* subsp. *carota*, *Artedia squamata* and *Echinophora trichophylla* more detailed investigations were conducted. Aiming to understand the reproductive system of the plant as a prerequisite for discussing the significance of the dark umbel centres, we studied architecture, andromonoecy, flower morph dimorphism, flowering sequence and insect visitor behaviour on the umbels.

We considered insects as potential pollinators when they were powdered with pollen and touched the receptive stigmas. We did not count the pollen load to determine pollinators, because this was beyond the scope of the present study.

Umbels are always terminal. The umbel at the tip of the main stem is the main umbel (abbreviated: M), whereas the umbels terminating lateral branches are counted after their branch order (abbreviated: I, II, III, …). This numbering is in accordance with Troll and Heidenhain (1951); Reuther and Claßen-Bockhoff (2010, 2013) and Ajani and Claßen-Bockhoff (2019, 2021), but differs from that used by Doust (1980). The latter named the umbel of first branch order a secondary order and the umbel of second order a tertiary umbel that is confusing in our view.

#### Daucus carota subsp. carota.

**Figure 2.**
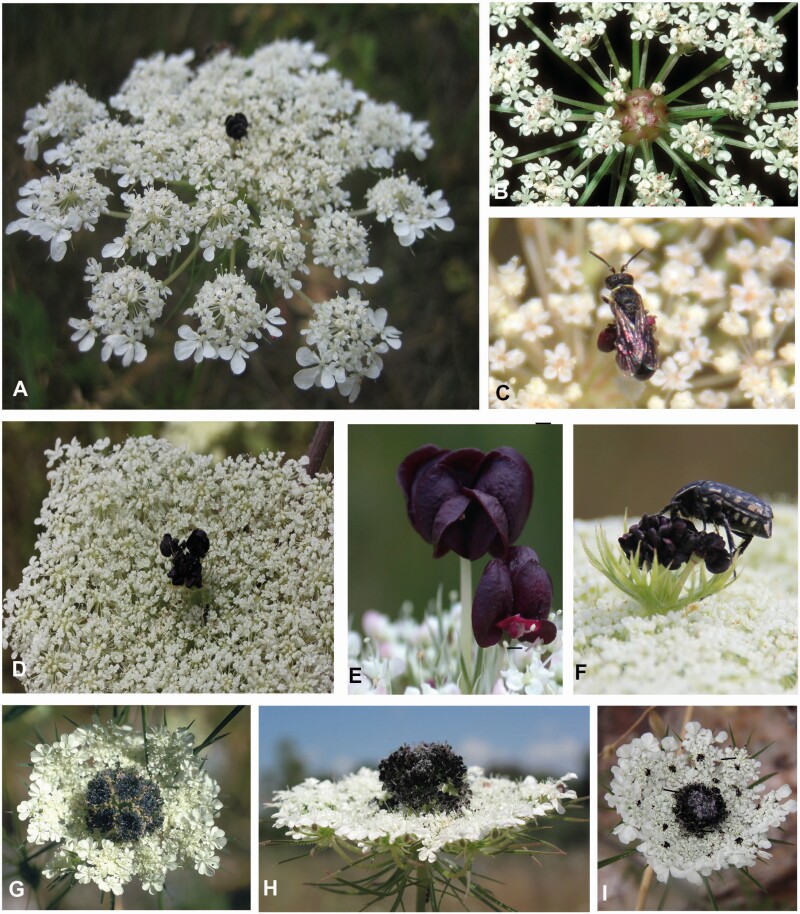
Dark umbel centres in the genus *Daucus*. (A-C) *D. carota* subsp. *carota.* (A) View onto an umbel with a red flower in its centre. (B) Rare case of an umbel with a swollen, reddish centre. (C) A male *Hylaeus* bee on the top of the dark flower. (D–F) *D. carota* subsp. *maximus.* (D) Huge umbel (diameter ≥ 20 cm) with a prominent dark flower. (E) Dark flowers protrude from the umbel plane. (F) A beetle inspecting the dark flower. (G and H) *D. bicolor* found at Side, S-Anatolia. Umbel from the top (G) and side view (H). The innermost umbellets form a dark-coloured conglomerate protruding from the white plane of the remaining umbellets. (I) *D. guttatus.* The central conglomerate is smaller and added by dark flowers in each umbellet. Population from Side, S-Anatolia.

The wild carrot is the most famous representative of the species producing dark-centred umbels (Fig. 2A). We analysed a huge natural population growing on the campus of the Johannes Gutenberg-University Mainz, Germany. The study was already conducted in summer 2003 and 2004, but only part of the results were published (Reuther and Claßen-Bockhoff 2010). We already confirmed modular growth, andromonoecy (60.6 %, *n* = 6) and multicyclic protandry with strict phase separation on the umbel level, but overlaps within the plant (see also Koul *et al*. 1989).

As the wild carrot is known for its labile expression of the red flowers (e.g. Daumann 1973), we labelled 104 individuals and recorded the appearance of dark-centred umbels in the population and their position within the individuals. Furthermore, we observed umbel visitors and their behaviour for 4 weeks.

#### Artedia squamata.

 Field investigations were conducted at the campus of the Middle East Technical University (METU), Ankara, Turkey. Several populations with hundreds of individuals are native to the area.

The architecture and organization of representative umbels were analysed and documented in side-views. A total of 20 individuals were labelled and daily observed to reconstruct the flowering sequence. Three flowering stages were distinguished: the staminate stage, in which anthers dehisce and release pollen, the sterile phase, in which the last pollen is shed and the styles start to elongate, and the receptive phase, in which styles are spread and pollen grains held by the wet stigmatic surfaces. Flower morphs can be best distinguished in the postfloral stage when the ovaries of the perfect flowers start to mature (Ajani and Claßen-Bockhoff 2019, 2021). Number and position of the perfect flowers were recorded in each umbel of 10 individuals. The degree of andromonoecy was calculated as the percentage of staminate flowers in a plant. Fruit set was determined by the number of mature fruits related to the total number of perfect flowers. Insect visitors, their frequency and behaviour on the umbels were documented by photos and videos during the flowering season (June) of three consecutive years (2014–2016).

Insects were determined using standard keys (Şengün and Bilige 1959). Only those insects visiting the staminate and receptive phase of the umbels and touching the reproductive surfaces were classified as potential pollinators. To test the effect of the dark brush on beetles, experiments were conducted with five beetle species considered to be pollinators. In the experiment, we had three treatments. For each, we selected a small, isolated patch of the population separated from the other two plots, and used all individuals (15–20). In the first treatment, we removed the dark brush in all umbels, in the second, we added two brushes to the central one, and in the third, the umbels were untreated serving as control. Before manipulating the umbels, we confirmed that all five beetle species were present on the patches. We then labelled 10 umbels from 10 individuals per treatment and recorded the beetle visits twice a day for 20 min on five consecutive days.

#### Echinophora trichophylla.

A natural population of this species was investigated in Küplüköy near Bilecik, NW, Anatolia. Architecture, degree of andromonoecy, flower morph distribution, flowering sequence and fruit set were analysed as in *A. squamata*. Insect visitors were observed and documented by photo and video in June 2015 and 2016.

### Umbel development and surface structures

Whereas *Daucus carota* presents red flowers in the umbel centre, the morphology of the peculiar dark structures in the other species is unknown. To test the hypothesis that these structures were reduced and modified umbellets (Cullen 1972; Eisikowitch 1980), we investigated umbel development in *Artedia squamata* and *Echinophora trichophylla.* Umbel buds of different ages were fixed in 70 % ethanol. The samples were dissected in the lab, dehydrated in an ascending alcohol series, critical point dried (BAL-TEC CPD 030) and sputtered with gold (BAL-TEC SCD 005) before they were interpreted and documented using the scanning electron microscope (XL-30 ESEM, Philips). All technical steps were conducted following the manufacturer’s protocol.

To test whether the micromorphology of the central structures differs from that of other umbel parts, we investigated the umbels of six species (*Daucus carota* subsp. *carota*, *D. carota* subsp. *maximus*, *Artedia squamata*, *Echinophora trichophylla*, *Tordylium aegyptiacum*, *T. cappadocicum*) using the SEM as described above.

## RESULTS

We found natural populations of eight apioid species (one of them represented by two subspecies) with dark-centred umbels, among them *Tordylium cappadocicum* as a new example (Table 1). All species grew in open, often ruderal areas or along roadsides, where they usually appear in large populations of several hundred to thousands of individuals.

**Table 1.** Species and localities included in the present study.

Species	Locality, characteristics, study time
*Daucus carota* subsp. *carota*	Biannual, erect, 100–150 cm • Germany, Aachen-Seffent; 169 m, ruderal place, July 1988
	• Germany, JGU Campus, Mainz. 120 m, June–July 2002, 2004
	• Turkey, Ayazma Spring Nature Park, Çanakkale, NW Anatolia. August 2014
*Daucus carota* subsp. *maximus*	Biannual, erect, up to 250 cm • Turkey, 35 km E of Antalya and 11 km W of Serik, S-Anatolia, ca. 100 m above sea level, June 2015 • Turkey, Antalya, Side, opposite to the entrance to the historic city, sea-level, June 2014, 2016, co-occurring with *D. guttatus and D. bicolor*
*Daucus bicolor*	Annual, erect, 80–100 cm • Turkey, Side, opposite to entrance to the historic city, S-Anatolia, sea-level, June 2014, 2016, co-occurring with *Daucus carota* subsp. *maximus* and *D. guttatus*
	• Turkey, Bilecik, Küplüköy village, NW Anatolia, 358 m, June 2014, 2015, co-occurring with *Daucus guttatus, Artedia squamata* and *Echinophora trichophylla*
*Daucus guttatus*	Annual, erect, 80–100 cm • Turkey, Side, opposite to entrance to historic city, sea-level June 2014, 2016, co-occurring with *Daucus carota* subsp. *maxima* and *D. bicolor*
	• Turkey, Bilecik, Küplüköy village, NW Anatolia, 358 m, June 2014, 2015, co-occurring with *D. bicolor*, *Artedia squamata* and *Echinophora trichophylla*
*Artedia squamata*	Annual, erect, 20–80 cm • Turkey, METU Campus, Ankara, C-Anatolia, 1000 m, *Stipa* steppe, May–July 2014, June 2015, 2016
	• Turkey, Karaman, ca 50 km S of Ermenek, Central Taurus, 900 m, Mediterranean vegetation along the road, June 2014
	• Turkey, SE Anatolia, Şanlıurfa: Karaköprü, among *Pistacia* gardens, ca. 700 m, in fields; co-occurring with *Tordylium cappadocicum*
*Echinophora trichophylla*	Perennial, erect, 100–150 cm, endemic • Turkey, Bilecik, Küplüköy village, NW Anatolia, 358 m, June 2014, 2015, co-occurring with *Daucus guttatus, D. bicolor* and *Artedia squamata*
*Dicyclophora persica*	Annual, up to 40–50 cm, endemic • Iran, Hormozgan province, N of Bandar Abbas, between Doroudi deviation and Shamil, frequently at the roadside, 915 m, April 2018
*Tordylium aegyptiacum*	Annual, erect, 25–80 cm • Turkey, SE Anatolia, between Osmaniye and Gaziantep, ca. 53–54 km before Gaziantep, 560 m, in roadsides, fields and waste places, April, 2016
*Tordylium cappadocicum*	Annual, erect, 25–50 cm, endemic • Turkey, SE Anatolia, Şanlıurfa: Karaköprü, among Pistacio gardens, ca. 700 m, in fields, co-occurring with *Artedia squamata*, May, 2016

### Diversity of dark-centred umbels

All species investigated had white umbels composed of many usually densely aggregated umbellets. During anthesis, the umbels are flat presenting a landing platform for all kinds of floral visitors. Conspicuous ray flowers appear in *Artedia squamata*, *Echinophora trichophylla* and *Dicyclophora persica*.

#### Daucus carota subsp. carota.

Dark flowers do not occur in all individuals of a population. Among the 104 labelled individuals, 72 % had red flowers, whereas the remaining plants were completely white-flowered. Interestingly, individuals with dark-centred main umbels did not present dark flowers in each umbel.

**Figure 3.**
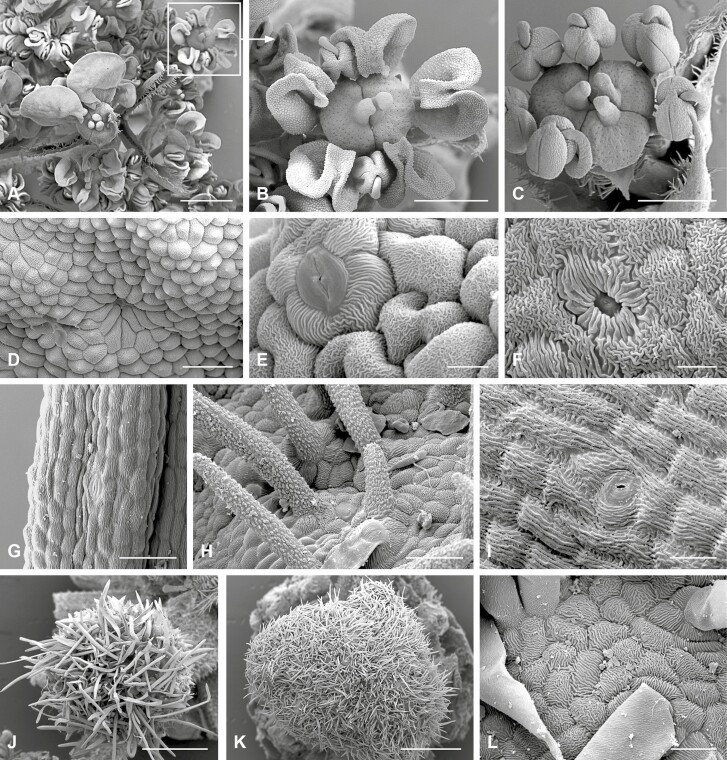
Flower dimorphism and micromorphological structures. (A–F) *Daucus carota* subsp. *carota*. (A) Central part of an umbel showing the enlarged flower in the centre. (B) Detail from A, showing a perfect flower in a peripheral position. The flower represents the typical pentamerous apioid flower with flexed petals alternating with stamens and with nectar-secreting stylopodia with two styles. (C) The terminal flower is not only larger than the lateral flowers but also often differs in number; here is an example with six stamens and three styles. (D–F) Stomata and cuticular structures are found on the abaxial petal side (D), the adaxial petal side (E) and the stylopode (F). (G) *Artedia squamata*. Detail of a single brush element showing the longitudinal epidermis cells and a stoma. (H and I) *Echinophora trichophylla*. (H) Trichomes with a specific cuticular surface on the middle part of the central plug. (I) Longitudinal epidermis cells with cuticular stripes and a stoma at the base of the central plug. (J) *Tordylium cappadocicum.* Tip of the central structure is covered with long hairs. (K and L) *T. aegyptiacum.* (K) The tip of the central plug is larger than in *T. cappadocicum*, and covered with shorter hairs. (L) Epidermis cells with smooth unicellular hairs. Bars: 1 mm (A, J and K). 500 µm (B and C). 50 µm (D, G and H). 20 µm (I and L). 10 µm (E and F).

If present, umbels produced 1–2 (−6) red flowers; however, in single first-order umbels, up to 18 red flowers were found. The central flowers were larger than the white flowers and often monstrous producing more floral organs and being exposed by a thick peduncle (Fig. 3A–C). However, they showed the same diversity of epidermal cell shapes, cuticular patterns and stomata found all over the umbel (Fig. 3D–F). The dark flowers varied considerably in size and colour ranging from almost black to light red. They were perfect and set fruit; only single flowers remained closed showing a cleistogamous behaviour.

Most individuals with dark-centred umbels were vigorous, early flowering plants (starting in early June) branched up to the third order. Individuals flowering from mid of July onwards were generally weaker than the first flowering plants and produced rarely dark flowers.

If a plant had a red flower in the main umbel, it most likely also produced dark flowers in the first-order umbels (74 %, *n* = 822). With increasing branch order, the percentage of dark flowers decreased from 23 % in the second (*n* = 2135) and 3 % in the third (*n* = 845) to 0 % in the fourth (*n* = 98) orders. As the averaged flower number per umbel (in 4 individuals) also decreased with umbel order from 1982 flowers in the main umbel to 1699, 1272, 893 and 177 flowers in the umbels of first to fourth order, the production of dark flowers is clearly correlated with umbel size and age.

The facultative formation of the red flower in the umbel centre was paralleled by the variable formation of a terminal flower in the umbellet centre. Among the test plants, 77 % of the individuals had closed umbellets (with terminal flowers) in all umbellets of the main umbel, 20 % in part of these umbellets and 3 % only open umbellets (no terminal flowers) throughout the main umbel. In the first-order umbels, the numbers were 15.6 %, 46.9 % and 37.5 %, in the second-order umbels 3.8 %, 11.5 % and 84.6 % and in the third-order umbels 0 %, 4.6 % and 95.4 %. The data clearly showed that terminal flower formation in the umbellets decreased with umbel order comparable to the decrease of red flower formation in the umbel centre.

Modified umbels were observed at a roadside in Aachen-Seffent, Germany. Within a large natural population, one individual plant produced umbels with swollen reddish centres (Fig. 2B). The innermost umbellets were affected by the swelling and gradually reduced.

The umbels of *D. carota* were frequently visited by insects. During our 4-week observation time, we recorded a total of 28 insect species from 18 families and 4 orders. The dominant groups were flies (10 sp.), bees (10 sp.) and beetles (7 sp.). Motivation and behaviour of the visitors were highly diverse. Most visitors fed from pollen and/or nectar. Beetles were waiting for a mating partner or, in the case of predatory species, for prey. As to the activity of the insects, either only few flowers (some Syrphidae), few umbellets (Cerambyciade, Scarabaeidae, Chrysididae) or all umbellets were visited and inspected (Alleculidae, Mordellidae, Chrysididae, Formicidae, Gasteruptiidae, Ichneumonidae, Calliphoridae, Muscidae, some Syrphidae). Some syrphids and other insects left the umbels after a few seconds whereas some beetles (mainly Cerambycidae, Scarabaeidae) stayed on the umbels for minutes or used the umbel as a permanent place (Mordellidae, Melyridae) only rarely switching to another umbel.

Whereas the insects observed at Mainz did not show a significant interest in the dark flowers, a clear biotic interaction was observed during a field trip to NW Anatolia. A large population of *D. carota* subsp. *carota* grew along a forest track at the Ayazma Spring Nature Park, Çanakkale. Insect visitation was generally low: only few individuals of different beetle, fly and bee species were seen during the 30-min observation time. An exception was made by male *Hylaeus* (Colletidae) individuals (easily recognizable by their white face mask), who appeared in high numbers and were present during the whole time. They flew directly and precisely to the dark centre and landed on the dark flower (Fig. 2C). Here, they inspected the flower for up to 10 s and, then, either flew away or started to feed pollen. Sometimes two or three individuals came at the same time chasing away each other. Some bees bent their abdomen as if they intended to copulate, but no attempts of copulation could be observed.

#### D. carota subsp. maximus.

The giant carrot (*D. carota* subsp. *maximus*) is a biannual plant and was found in June at two localities in S-Anatolia (Turkey), one at a roadside above Alanya and the other one on a ruderal area close to Side. Individuals varied from 100 to 200 cm in height (Table 1).

Umbellets were densely aggregated and formed a flat plane of a large diameter (≥20 cm; Fig. 2D). One or few dark flowers protruded from the umbel plane and were highly conspicuous (Fig. 2E). They were stalked by thick peduncles that were occasionally united to a single thick structure. Visitor diversity appeared to be not as high as in *D. carota* subsp. *carota*. We observed predominantly beetles (*Oxythyrea funesta*, *Cantharis* sp., *Rhagonycha fulva*, *Mylabris* sp.) feeding pollen and waiting for a mating partner. Single beetles were particularly attracted by the dark flower(s) and carefully inspected them (Fig. 2F), before turning away to start feeding.

#### Daucus bicolor and D. guttatus.

 Two more *Daucus* species were found at various places in S and NW Anatolia (Table 1). Both species had very conspicuous umbels of moderate size. The innermost umbellets formed a dense conglomerate of dark flowers protruding from the white plane of the remaining umbel.

In *D. bicolor*, up to eight (rarely more) umbellets were integrated into the dark centre. They were easily recognizable as the outermost flowers of the umbellets tended to become brownish (Fig. 2G and H). The umbels of *D. guttatus* had smaller umbellet aggregates in the umbel centre compared to *D. bicolor*, but presented additional dark flowers in the centre of each umbellet (Fig. 2I). Thereby, a spotted pattern resulted resembling an umbel with less aggregated umbellets. In some umbels, the colouring was irregular, for example, radial sectors of the umbels were dark coloured. The species co-occurred with *D. bicolor* at Side (S-Anatolia) and at Küplüköy (NW Anatolia), where also *A. squamata* and *E. trichophylla* were co-flowering. Whereas the population observed at Side had black-centred umbels in all individuals and umbels, completely white individuals were seen in the NW population.

In both species, terminal flowers and umbellets were lacking. The innermost umbellets set fruit as the outer ones. Their flowers were dark coloured and covered with white hairs giving them a bluish appearance. A range of insects, predominantly beetles, were observed visiting the umbels. They showed a similar diversity in behaviour and motivation as the insects observed on *D. carota* subsp. *carota*.

#### Artedia squamata.

**Figure 4.**
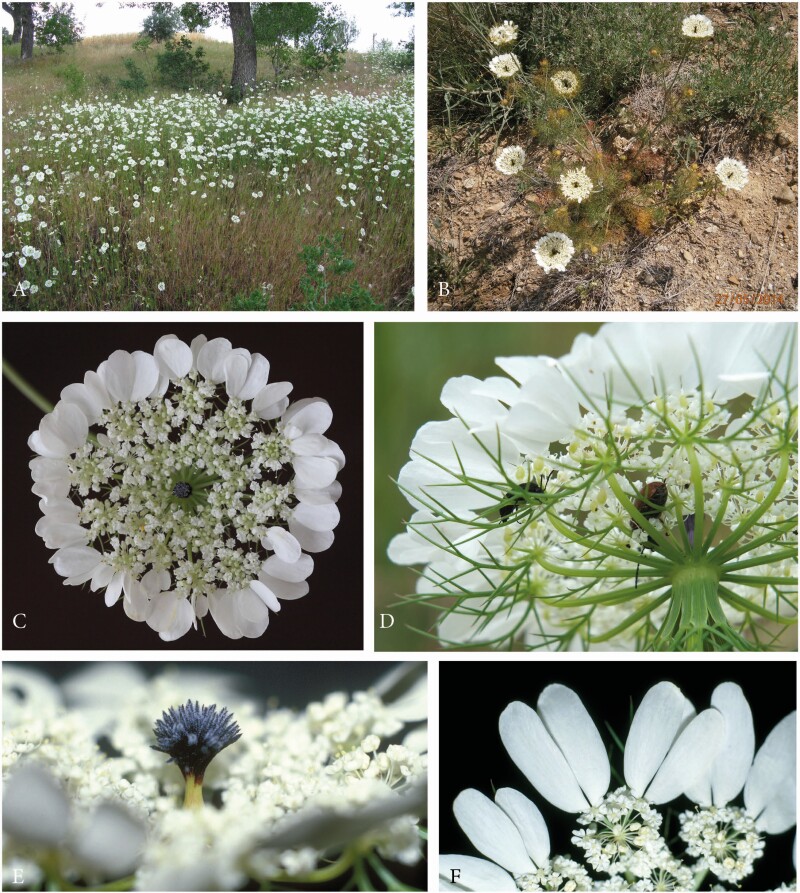
Artedia squamata. (A) Natural population, METU Campus, Ankara, Turkey. (B) Individual plant with a main umbel and several lateral umbels of first order. (C) Umbel from the top view showing the arrangement of the ray flowers. (D) Umbel from below with pinnate involucral bracts. (E) Brush-like structure in the umbel centre. (F) Each ray flower forms two enlarged asymmetric petals that are mirror images of each other.

*A. squamata* is an annual with conspicuous white umbels. The species was predominantly studied on the METU Campus in Ankara, Central Anatolia (Fig. 4A, Table 1).

Umbels were white, flat and surrounded by large ray flowers. Umbellets were densely aggregated in the umbel centre, but more loosely arranged at the periphery, where some beetles crawled to the lower side for resting (Fig. 4C and D). Each of the outermost umbellets formed two or three ray flowers. The two lobes per ray flower came from two neighboured petals that were asymmetrically enlarged and mirror images to each other (Fig. 4F). The umbel centre was dominated by a prominent dark brush-like structure (Fig. 4E). This structure had a column-like base and a tuft composed of filamentous elements. Though these elements were usually black, they appeared bluish or silvery due to their dense cover with white hairs.

Size, colour and hairiness of the brush-like structures varied considerably. These were up to 5 mm long and usually protruded from the umbel plane in the main umbels; they decreased in size in higher-order umbels. Column and tuff had the same dark, reddish or rarely white colour or the base was greenish and only the tuff was dark. Hairs usually formed a dense coverage, but were sometimes lacking.

#### Echinophora trichophylla.

**Figure 5.**
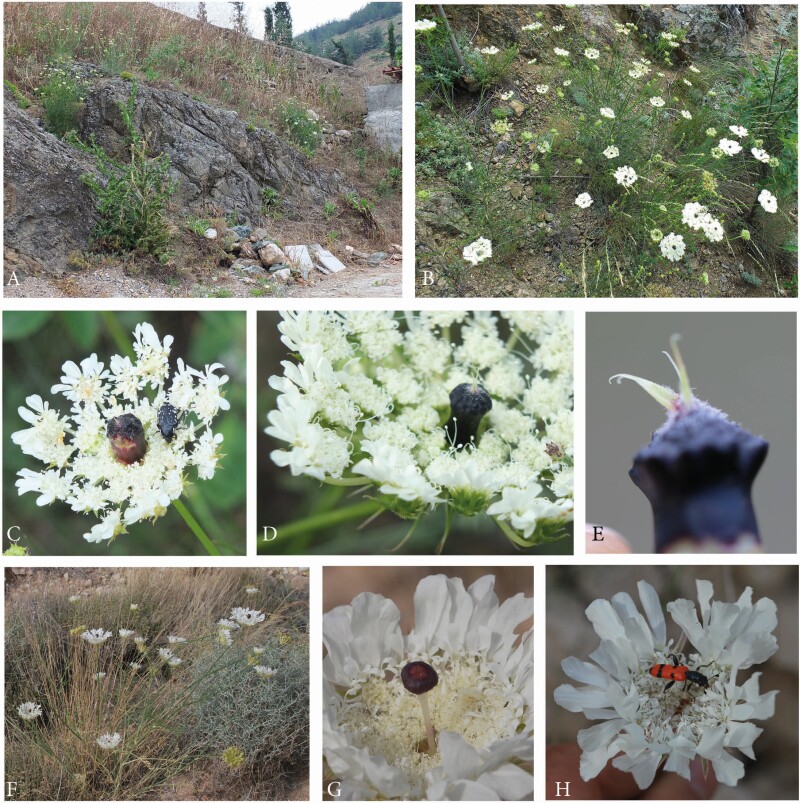
Representatives of the Echinophoreae. (A–E) *Echinophorea trichophylla*. (A) Natural locality close to Küplüköy village, NW Anatolia. (B) Flowering individuals. (C and D) Umbels with central plug-like structures of variable size and shape. (E) Central structure; note the occasionally appearing bracts on the top of the plug. (F–H) *Dicyclophora persica*. (F) Individual plant at a natural locality north of Bandar Abbas, South Iran. (G) Dark-red structure in the umbel centre. (H) Umbel from above with a *Trichodes* beetle (Cleridae).

This species was only found at a single locality in NW Anatolia (Table 1). Plants had densely aggregated umbels with ray flowers at the outermost umbellets (Fig. 5C and D). Each ray flower had three enlarged petals, a bilobed one in the middle flanked by a one-lobed petal at each side. The peripheral promotion of the ray flowers had its counterpart in the outer involucral bracts which were likewise enlarged. A massive dark plug stood in each umbel centre. It was either egg-shaped (Fig. 5D) or cylindrical with a broad rim at its top (Fig. 5E). The uppermost part was often covered with white hairs; occasionally, bract-like structures appeared on top of the plug (Fig. 5E). Size and colour varied among individuals.

#### Dicylophora persica.

*D. persica* is an annual, endemic species found in S Iran. Individuals grew along roadsides, flowered in March and were visited by beetles and other insects (Fig. 5F and H). The umbels resembled those of *Echinophora trichophylla* in being densely aggregated, surrounded by ray flowers (larger than those of *Echinophora*) and characterized by a dark structure in the umbel centre. These structures clearly protruded from the umbel plane (Fig. 5G). They were usually club-shaped with a white, long and thin column and a purple hood. However, the structure varied in shape, colour and size. The tip was globular to fusiform and sometimes even reduced.

#### Tordylium aegyptiacum and T. cappadocidum.

 Among the genus *Tordylium*, at least two species produce dark-centred umbels *T. aegyptiacum* and *T. cappadocicum* were studied in SE Anatolia (Table 1).

**Figure 6.**
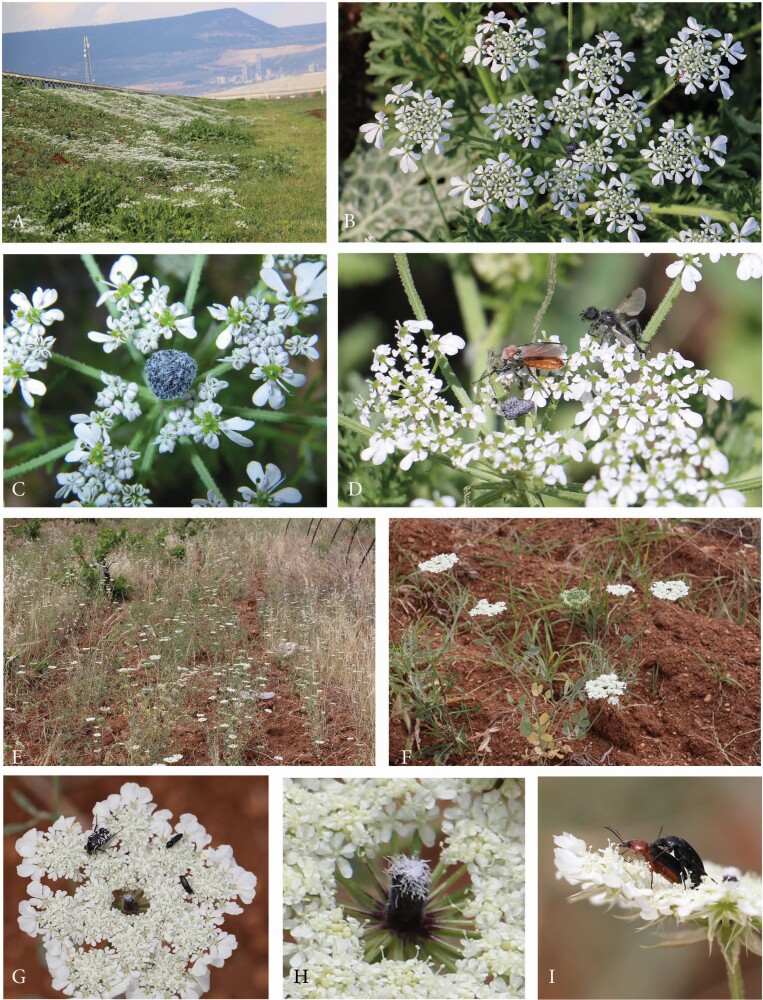
Dark-centred umbels in the genus *Tordylium*. (A–D) *T. aegyptiacum.* (A) Large population between Osmaniye and Gaziantep, SE Anatolia. (B) Umbel with loosely arranged umbellets, moderately enlarged ray flowers and a less conspicuous, coloured centre. (C) Dark, T-shaped structure in the umbel centre, its flat top covered with white hairs. (D) Among the many insect visitors, fly species appeared to dominate. (E–I) *T. cappadocicum*. Natural population at Karaköprü, SE Anatolia. (F) Annual plant with the main umbel in the fruiting stage and five flowering umbels of first branch order. (G) The densely aggregated umbellets form a common plate for insect visitors. (H) Massive plug-like structure in the umbel centre, its tip covered with white hairs. (I) Beetles appeared to be the main pollinators.

*Tordylium aegyptiacum* had umbels with loosely aggregated umbellets (Fig. 6B). Ray flowers were present, but relatively small and inconspicuous. The dark structure in the umbel centre resembled the club-shaped structure in *Dicyclophora*. However, it was less conspicuous because the column was shorter and the flat hood densely covered with white hairs reducing the colour contrast to the white flowers (Fig. 6C). Umbels were visited by a broad range of insects (Fig. 6C and D), mainly flies from the families Syrphidae, Calliphoridae (*Lucilia* sp., *Calliphora* sp.) and Tabanidae. More rarely, beetle species from the families Cantharidae, Coccinelidae (*Coccinella septempunctata*) or Scarabaeideae (*O. funesta*), bees (*Apis mellifera, Anthophora* spec.) and butterflies (*Vanessa cardui)* were observed feeding and/or mating on the umbels.

**Figure 7.**
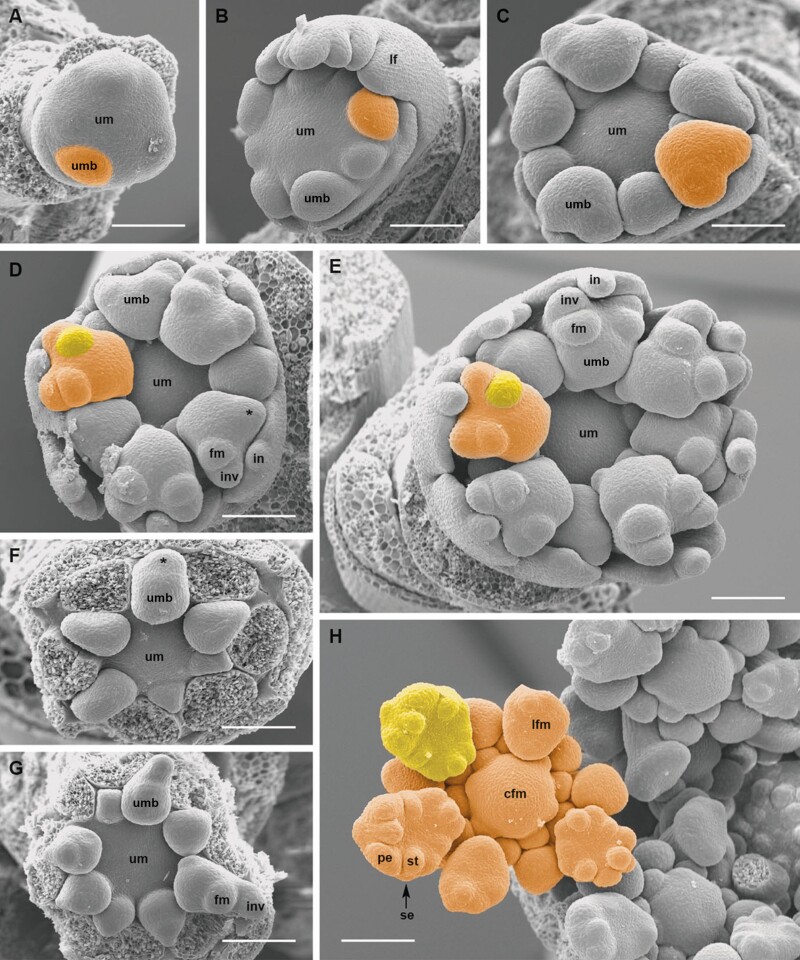
*Artedia squamata.* Umbel and umbellet development. (A) Umbel meristem (um) with first umbellet meristems (umb). (B) Spiral initiation of outer umbellet meristems. lf, leaf. (C) Eight outer umbellets were initiated, the oldest ones in the bilobed stage. (D) The two lobes of the umbellet meristems merge each into a bract-flower meristem unit (*). fm, flower meristem. in, involucral bract. inv, involucellar bract. (E) With the ongoing expansion of the umbel meristem, the inner umbellets become initiated. (F and G) Developing inner umbellets (outer ones removed); the centre of the umbel meristem remains naked. (H) Umbellet with developing flowers, the central flower meristem (cfm) larger than the inner lateral flower meristems. (lfm). pe, petal. se, sepal. st, stamen. Bars: 100 µm. All figures in the same scale.

*Tordylium cappadocicum* is endemic to Turkey and here described for the first time as a species with dark-centred umbels. Individuals differed from *T. aegyptiacum* in several characters. First, the umbels were densely aggregated (Fig. 6G), second, the central structure was plug-shaped as in *Echinophora trichophylla* (Fig. 7H) and, third, the main visitors were beetles (Fig. 6I) followed by a few fly and bee species. Two beetle species were permanently present on the umbels. Insects were mostly resting next to the dark plugs where crab spiders were hidden hunting pollinators and visitors.

In the area, *Tordylium* species co-occured with *Artedia squamata*. As in this species, the innermost umbellets pointed outwards, so that their peduncles could be seen from above (Figs 4C and 6H). These were green or black like the centre and, then, increased the visual effect of the dark centre. In any case, they formed a cavity that was frequented by beetles for sleeping or reaching the lower side of the umbel. This behaviour was particularly common at noon (between 12:00 and 15:30) when the activity of the insects declined.

### Developmental of central structures and micromorphological patterns

**Figure 8.**
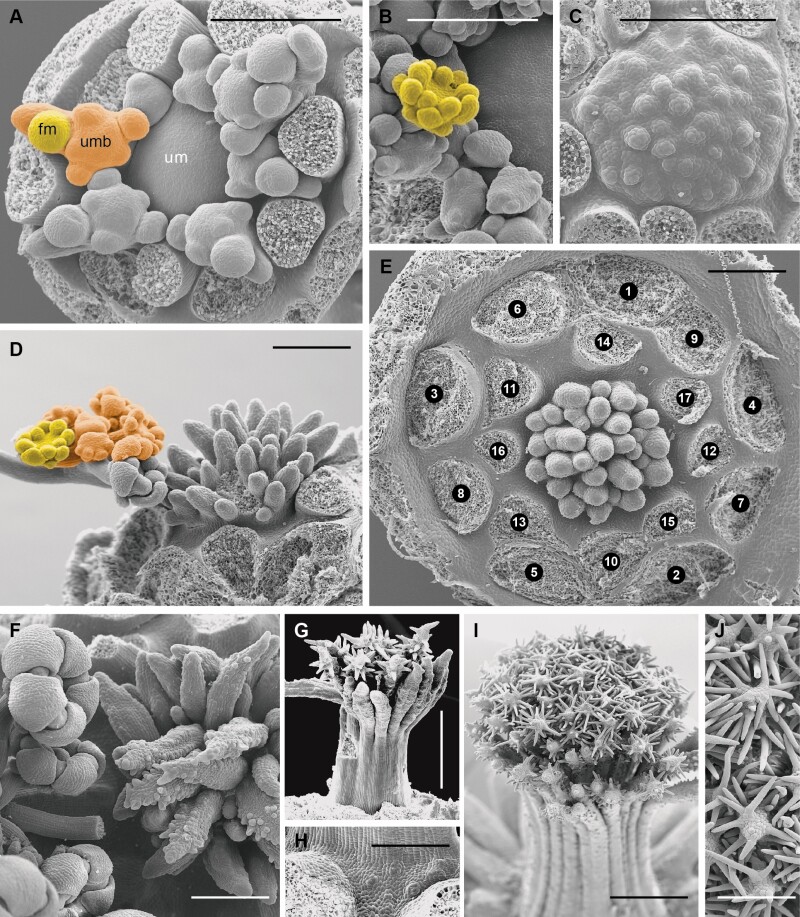
*Artedia squamata*. Development of the central brush. (A) The central part of the umbel meristem (um) is still in the naked stage when the innermost umbellets (umb) fractionate flower meristems (fm). (B) When the flowers of the innermost umbellets fractionate floral organs, the centre of the umbel meristem starts fractionating. (C) The fractions of the umbel centre appear in an irregular order. (D) The fractions cover the whole centre and elongate; a weak centrifugal development is recognizable. (E) View onto the umbel centre with all umbellets removed to show the distinct border between the umbellets and the central fractions. Numbers indicate the spiral sequence of umbellet initiation (from old to young). (F) When the innermost flowers are in the bud stage, the fractions start to produce hairs in a centrifugal direction. (G) The umbel receptacle elongates providing a common base for the fractions; hair formation continues. (H) At the base of the elevating receptacle, stomata appear. (I and J) The adult brush is composed of the common base and free arms completely covered with long unicellular hairs sticking out in all directions. Bars: 200 µm (A–F, H and J), 500 µm (G and I). Note that A–C are at the same scale and G and I as well.

**Figure 9.**
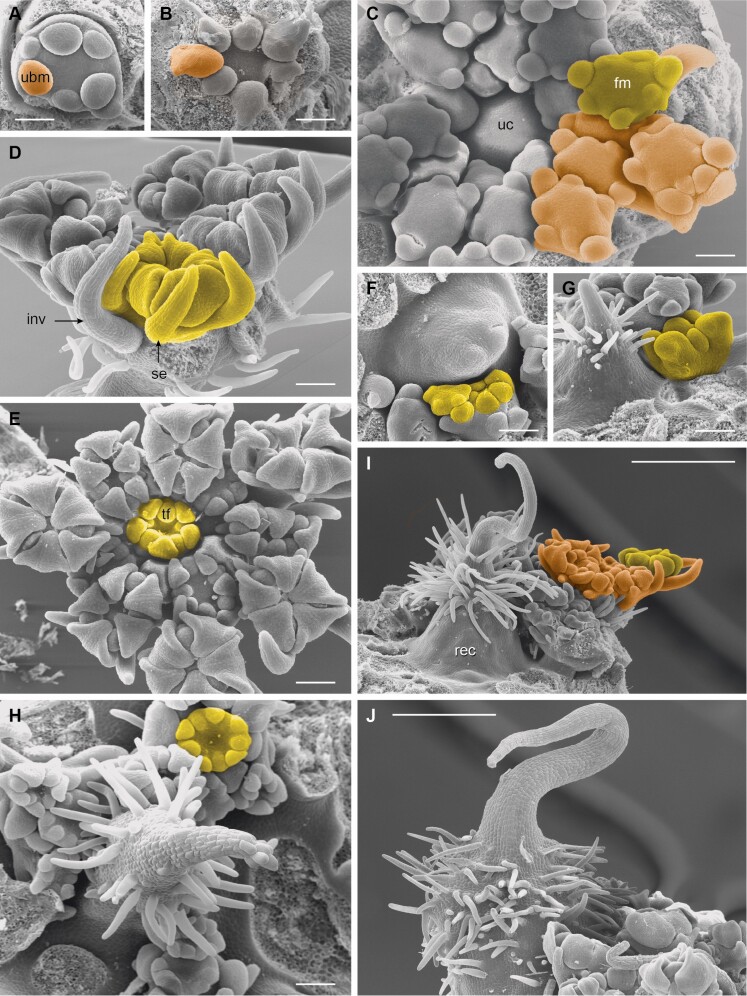
*Echinophora trichophylla.* Umbel development. (A) Umbel meristem with first umbellet meristems (ubm). (B) View onto the inner umbellet meristems (outer ones removed) showing the naked umbel centre. (C) The umbel centre (uc) starts bulging when the flower primordia (fm) of the inner umbellets fraction floral organs. (D) Umbellet in the prefloral stage. inv, involucellar bract. se, sepal. (E) Top view of an umbellet showing the precurrent development of the terminal flower (tf). (F and G) Elevation of the receptacle forming the central plug. (H–J) Central structures with a massive base, hairy zone and apical bract-like structure. Bars: 100 µm (A-H), 500 µm (I and J).

The umbels of *Artedia squamata* and *Echinophora trichophylla* develop from rather large reproductive meristems. Each umbel meristem fractions umbellet meristems, which fraction flower meristems in a second step (Figs 7A–C and 9A–C). The process starts at the periphery with a fast spiral initiation of the outermost umbellets and proceeds in centripetal direction. During fractionation, the meristem enlarges providing new space for the more inwards placed umbellets developing with delay (Fig. 7D–H). Interestingly, in both species, the central area of the umbel meristem remains undifferentiated (Figs 8A and B and 9C and G).

In *Artedia squamata*, the meristem centre remains naked for a long time. When the outermost umbellets initiate flower organ primordia and the innermost umbellets start to fraction (Fig. 8B), the first structures appear in the umbel centre (Fig. 8C). These structures are irregularly arranged bumps elongating to finger-like tubes of different length (Fig. 8C–E). In a later stage, their surface becomes covered with white spreading hairs (Fig. 8F–J) and the base of the whole cluster elongates in a column-like manner (Fig. 8G). The column is striped indicating the congenitally united tubes. In the adult stage (Fig. 8I), stomata appear around the base of the column (Fig. 8H) and the bristles of the brush.

In *Echinophora trichophylla*, the meristem centre remains naked and starts to bulge when the flowers of the innermost umbellets start to initiate floral organ primordia (Fig. 9A and C). No individual effigurations appear, instead the bulge elongates as a whole and forms a broad plug (Fig. 9F). The plug either ends roundish and is completely covered with long white hairs or it bears a filiform tip protruding from the hairy zone (Fig. 9F–H). The tip resembles a bract (Fig. 9D: inv) in size and slender shape.

As to the surface patterns, no obvious differences were found between peripheral and central umbel structures. In all species investigated, the epidermis cells showed similar cuticular stripes (Fig. 3E, F, I and L). They were round or elongated depending on the development of the underlying structure. Except *Daucus carota,* which had papillate cells on the abaxial and bulged cells on the adaxial petal side (Fig. 3D and E), the cells were flat. Epidermal hairs were unicellular and smooth and only in *Echinophora trichophylla* covered with cuticular nodules (Fig. 3H and L). The appearance of the hairs was closely linked with the dark central structures, which got a complete or partial white coat by the hairs. Only in *Daucus carota*, the central flowers had a smooth surface (Fig. 2E). Stomata were found in all species, predominantly on the dark structures irrespective of whether these were flowers (*Daucus*) or receptacle excrescences. They were anomocytic or diacytic and regularly embedded in the epidermis (Fig. 3D–G and I).

### Case studies

To elucidate the functional significance of the dark umbel centres, knowledge about visitors versus pollinators and the motivation and behaviour of the insects is needed. Given that the investigated plant species are self-compatible, andromonoecious and protandrous, additional information about their architecture and flowering sequence is needed to distinguish potential pollinators from visitors and to understand the reproductive system of the plants. Part of these data was collected in *Artedia squamata* and *Echinophora trichophylla*.

#### Case study I.

*Artedia squamata* is a common annual species flowering from the end of May to mid of July along roadsides, fields and open areas in scrubs and forests. We saw large and dense populations with several hundred or even thousand individuals at different places in the Taurus mountains up to 1600 m (Fig. 4A). The main study area was the protected *Stipa* steppe on the campus of the METU Ankara, where we conducted fieldwork in three consecutive years (2014–2016).

**Figure 10.**
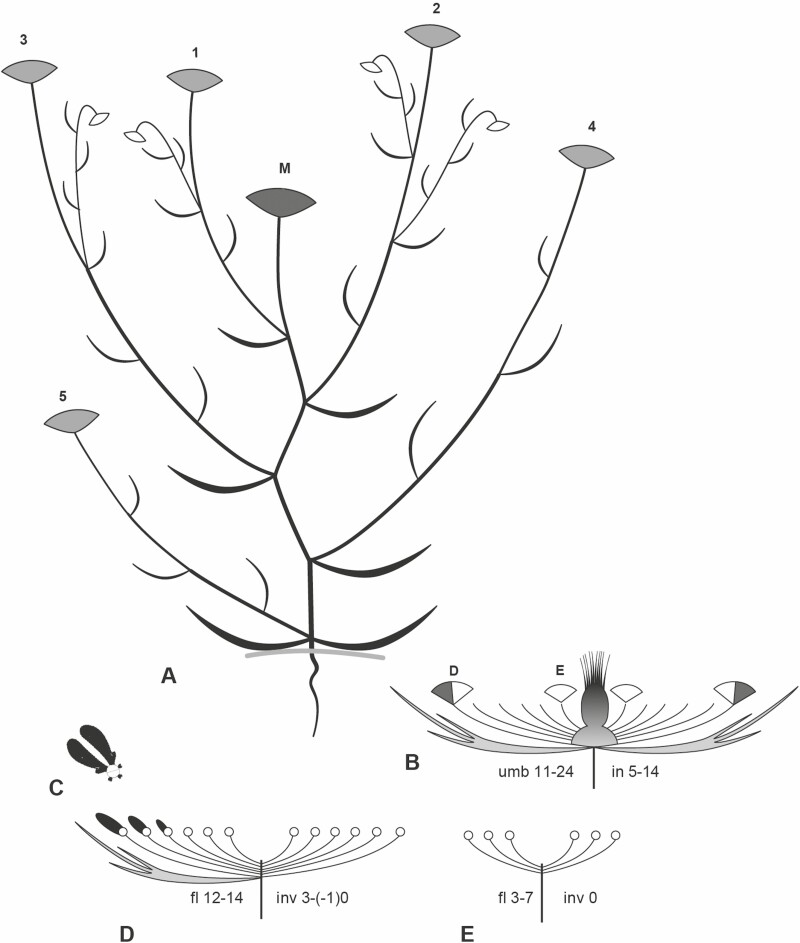
*Artedia squamata* Architecture and umbel construction. (A) Side-view of a representative individual with a main umbel (M) in fruit stage (dark grey), five flowering umbels of first branch order (grey, 1–5) and four umbels of second order in the bud stage (white). (B) Side-view of an umbel with the prominent brush in the centre. Perfect flowers (dark grey) only appear at the outer side of the peripheral umbellets. Only few umbellets are delineated, D and E refer to details given in D and E. in, number of involucral bracts. umb, number of umbellets. (C) Two asymmetrical ray flowers forming a bilobed structure. (D) Side view of a peripheral umbellet. fl, number of flowers. inv, number of involucellar bracts. (E) Side view of a completely staminate and bractless inner umbellet.

Individual plants reached a height of 20–80 cm. They had a dominant main axis bearing the main umbel and 2–7 lateral branches of first and few of second order each terminating in an umbel (Figs 4B and 10A). Only vigorous individuals were branched up to the second order.

**Figure 11.**
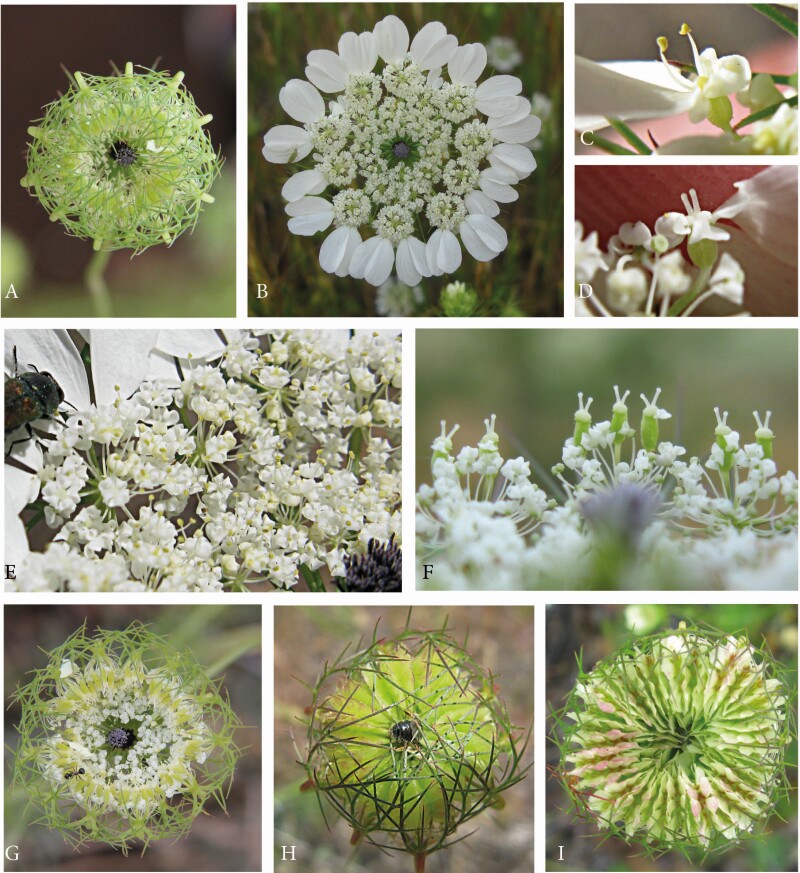
*Artedia squamata.* Life cycle of an umbel. (A) Prefloral stage. The involucral bracts protect the young flowers, the dark centre is already present. (B) Early staminate phase. (C) Ray flower presenting pollen; the styles are not yet elongated. (D) Ray flower in the receptive stage. (E) Umbel in the staminate flowering phase; all flowers bloom almost simultaneously. (F) Umbel in the receptive stage; note that only the peripheral flowers are perfect; in this stage, the large petals of the ray flowers are already shed. (G) The postfloral phase starts with the closure of the involucellar bracts. (H) The involucellar bracts become dry and hold the developing fruits. (I) The large and flat fruits open the involucral cage by physical pressure.

Umbels had an average diameter of 60 mm (*n* = 23). The main umbels were slightly larger (65 mm, range 52–79) than the first- (56 mm, range 47–70) and second-order umbels (60 mm, range 53–71). The number of umbellets per umbel varied between 11 and 24, and the number of flowers per umbellet was between 10 and 14 (Table 2). The average number of flowers per plant was 1094 (range 522–1455). Though the flowers were spirally initiated, usually three circles of flowers were recognizable. With few exceptions, only the outermost circle presented ray flowers (Figs 4F and 11B).

**Table 2.** *Artedia squamata*. Architecture and degree of andromonoecy. fl, flower. p, perfect flower. s, staminate flower. M, main umbel. umb, umbellet. um, umbel, I, first-order umbels, II, second-order umbels.

	Plant							Average	
Umbel	1	2	3	4	5	6	7	Total	%
M, s	169	189	226	185	209	142	161	183	78
M, p	56	46	59	38	65	41	49	51	22
M ∑ fl	225	235	285	223	274	183	210	234	
M ∑ um	1	1	1	1	1	1	1	1	
M ∑ umb	17	19	24	22	24	18	16	20	
I, s	443	509	381	307	312	140	128	317	78
I, p	151	143	95	76	89	40	40	91	22
I ∑ fl	594	652	476	383	401	180	168	408	
I ∑ um	3	3	2	2	2	1	1	2	
I ∑ umb	44	50	39	31	33	15	13	32	
II, s	492	383	561	409	312	216	110	355	78
II, p	144	106	149	94	93	67	32	98	22
II ∑ fl	636	489	710	503	405	283	144	453	
II ∑ um	4	3	4	3	3	2	1	3	
II ∑ umb	47	43	56	42	33	23	11	36	
∑ s	1104	1081	1168	901	833	498	399	855	78
∑ p	351	295	303	208	247	148	123	239	22
∑ fl	1455	1376	1471	1109	1080	646	522	1094	

##### Andromonoecy:

Umbels were andromonoecious producing staminate and perfect flowers. Interestingly, only the peripheral flowers of the umbel, that is, the peripheral flowers of the peripheral umbellets, were perfect (Fig. 11F). These flowers corresponded to the ray flowers. As the entire centre was staminate, the fruits had enough space to develop a considerable size (ca. 1 cm, Fig. 11I). The percentage of staminate flowers per plant was on average 78 % with no differences between the umbel orders (Table 2).

##### Flowering sequence:

Anthesis started with the opening of the main umbel. In the prefloral stage, the outermost umbellets were bent inwards allowing the pinnatified involucral bracts to form a protective cage (Fig. 11A). In the open umbel, all umbellets were almost in the same developmental stage. They flowered in a centripetal order; terminal flowers were lacking (Fig. 11B).

All flowers in an umbellet and all umbellets in an umbel were synchronized rendering the umbel staminate in the early and receptive in the later stage of anthesis (Fig. 11E and F). First, the perfect flowers at the outer margin of the umbel started to present pollen (Fig. 11C). They were followed by the staminate flowers which likewise needed a day to release the pollen. Then, after a sterile phase of 1 or 2 days (Table 3), in which the styles elongated (Fig. 11D), all perfect flowers entered the receptive phase at the same time. This phase was characterized by spread styles and wet stigmas visible by their shiny, drop-like exudate (Fig. 11D and F). The ray flowers started to drop down their enlarged petals rendering the umbel less attractive (Fig. 11F). At the end of anthesis, the outer umbellets bent inwards and the involucral bracts protected the ripening umbel until the mature fruits forced the cage open (Fig. 11G–I). Fruit set was over 95 % with 74 % well-developed (two mericarps) and 22 % weakly developed fruits (only one mericarp matured).

**Table 3.**
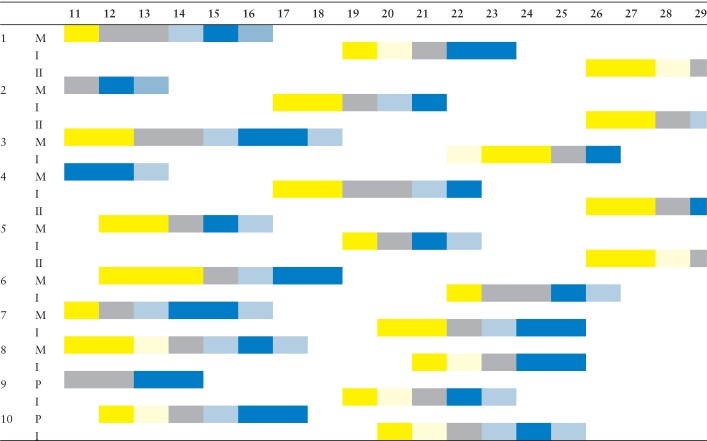
*Artedia squamata*. Flowering sequence of ten plants (1–10) from July 11 to July 29, 2014, at the METU Campus, Ankara. The small sampling already illustrates the strict phase separation within and the high phase overlap among plants. M, main umbel. I, all umbels of the first order. II, all umbels of second order. Blue, receptive flowering phase. Grey, sterile phase. Yellow, staminate flowering phase. Light colours indicate transitional stages.

Anthesis of a single umbel lasted 4–8 days (Table 3). When the main umbel started to fruit, all umbels of the first branch order repeated the protandrous behaviour in a synchronized way. When the first-order umbels were fruiting, all second-order umbels started to flower. The flowering sequence was, thus, strictly ordinal with no flowering phase overlap within or among umbel orders (Table 2).

In consequence, the plant was temporarily dioecious. Due to the large population size and the asynchronous flowering of neighbour plants, staminate and receptive phases were always present at the same time.

##### Umbel visitors and potential pollinators:

**Figure 12.**
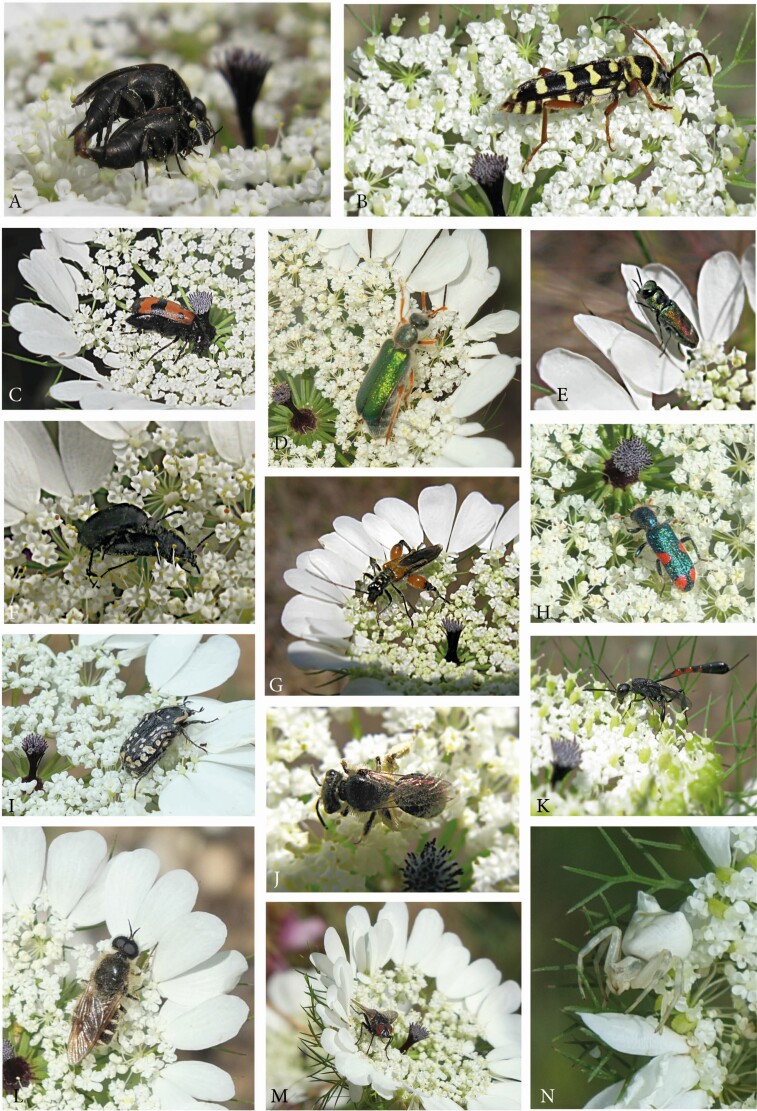
*Artedia squamata*. Umbel visitors. (A–I) Beetles (Coleoptera). (A) *Hoshihananomia* sp. (Mordellidae). Copulation. (B) *Plagionotus floralis* (Cerambycidae) on an umbel in the receptive stage. (C) *Mylabris quadripunctata* (Meloidae). (D) *Cercoma* sp. (Meloidae). (E) *Anthaxia* sp. (Buprestidae). (F) *Podonta* sp. (Alleculidae). Copulation. (G) *Oedemera brevipennis* (Oedemerideae). (H) *Trichodes quadriguttatus* (Cleridae). (I) *Oxythyrea funesta* (Scarabaeidae). (J and K) Bees (Hymenoptera). (J) *Dufourea* sp. (Apidae). (K) *Gasteruption assecator* (Gasteruptiidae). (L and M) Flies (Diptera). (L) *Lasiopa* sp. (Stratomyidae). (M) Muscidae. (N) Spiders (Arachnidae). *Thomisius* sp. (Thomisidae).

During the 2-month observation time in 2014, more than 50 visitor species were observed (Tables 4 and 5). The largest group was represented by beetles with 26 species from 12 families (Fig. 12A–I). Furthermore, 22 insect species from 9 orders and 3 spider species could be recognized (Fig. 12J–N).

**Table 4.** *Artedia squamata*. Beetles as umbel visitors and assumed pollinators in June–July 2014. METU campus, Ankara. mov., **movement** on umbels: f, fast. s, slow. +, often. -, rare. behav., **behaviour**: ag, aggressive. es, escaping. un, uninvolved. **reward**: ne, nectar. po, pollen. bold: high amounts. po.dep., **pollen deposition** on beetle body: el, elytytra. fe, femur. st, sternum. total, all body parts. **function**: **p+**, possible regular pollinator. p, possible pollinator. (p), possible occasional pollinator. v, visitor.

Family, genus, species	Motivation	mov.	behav.	reward	po. dep.	function
Buprestidae						
*Anthaxia* sp.	Food, copulation	f+	es	**po, ne**	el, st	p+
Cerambycidae						
*Leptura* sp.	Food	f+	un	**po**	el	(v)
*Plagionotus floralis*		s−	ag	po, ne	total	**p+**
*Pseudovadonia* sp.		s+	un	**po**	el, fe, st	p
Chrysomelidae						
*Bruchidius* sp.	Food, protection	s−	un	**po**	–	p
Cleridae						
*Trichodes quadriguttatus*	Food, perch	f+	ag	**po**	el, st	(v)
Coccinellidae						
1–2 species	Protection, perch	s−	un	–	–	v
Curculionidae						
≥2 species	Food, protection	s−	un	po	–	(p)
Melachiidae						
*Cordylepherus viridis*	Food	f+	un	**po, ne**	st	(p)
Meloidae						
*Cercoma* sp.	Food, perch	s−	es	**po**	el, st	p
*Mylabris quadripunctata*	Food	s−	un	po, ne	total	p+
*Mylabris* sp.						
*Oenas crassicornis*	Food, protection	f+	es	po		(p)
Mordellidae						
*Hoshihananomia* sp.	Food, copulation and protection	s+	un	po	total	**p+**
*Mordella* sp.		f+	es	**po**	el, fe, st	p
*Mordellistena*		f−			el, st	(p)
*Mordellistenula* sp.		f+			el, fe	v
*Mordellochroa* sp.	Food, protection				el, st	p
*Variimorda* sp.					el, fe, st	(p)
Oedemeridae						
*Oedemera brevipennis*	Food	s+	un	**po**	–	p
Scarabaeidae						
*Oxythyrea funesta*	Food	s+	un	po, ne	el, st	p
*Phylloperta horticola*				**po, ne**	el, fe, st	p
Tenebrionidae						
*Omophlus lepturoides*	Food, protection	s+	un	po	total	p
*Podonta* sp.	Food, copulation and protection			**po, ne**	el, st	**p+**

**Table 5.** *Artedia squamata*. Insects (except beetles) and spiders observed on umbels in June–July 2014. METU campus, Ankara. mov., **movement** on umbels: f, fast. s, slow. var, variable. +, often. -, rare. behav., **behaviour**: ag, aggressive. es, escaping. un, uninvolved. **reward**: ne, nectar, po, pollen, bold: high amounts. po.dep., **pollen deposition** on insect body: fe, femur. **function**: **p+**, possible pollinator. v, visitor.

Genus, species	Motivation	mov.	behav.	reward	po. dep.	function
Bees (Hymenoptera)						
*Ammophila* sp.	Food, perch	f+	un	ne		v
*Bombus* sp.	Perch	var.		–		v
*Dufourea* sp.	Food, protection	f+		**po, ne**	fe	**p+**
*Gasteruption assecator*	Food			**ne**		v
Bugs (Heteroptera)						
*Aelia acuminata*	Food, copulation and protection	s−	un	phloem		v
*Capicornis fuscispinus*						
*Graphosoma lineata*						
*G. semipunctatum*						
*Rhynocoris iracundus*	Food, perch, and protection		ag			
Cicada (Hemiptera)						
1 spec.	Perch, protection	s−	es			v
Butterflies (Lepidoptera)						
*Melanagria galathea*	Perch	s−	es			v
*Satyrium acaciae* S. spec.						
Grasshoppers (Orthoptera)						
*Chortippus* sp.	Food	s−	un	leaves, stems and flowers		v
*Oedipoda cerulescens*						
*Isophya* sp.						
*Poecilimon* sp.						
*Saga pedo*	Perch	s+				
Flies (Diptera)						
Asilidae—1 spec.	Food, perch	f+	un	insects		v
Mantids (Dictyoptera)						
*Mantis religiosa*	Food, perch, and protection	var.	ag	insects		v
Dragonflies (Odonata)						
*Sympetrum* sp.	Perch	f+	un			v
Antlions (Neuroptera)						
*Palpares libelluloidea*	Perch, protection	f+	un			v
Spiders (Arachnida)						
*Agelana labyrinthica*	Food, perch and protection	s−	ag	insects		v
*Thomisus onustus*						
Gnaphodisae—1 spec.		f−				

Insect motivation, behaviour and activity were highly diverse. Motivation ranged from feeding pollen and/or nectar to waiting for a mating partner or for prey and seeking a shady and save place for sleeping. Behaviour was aggressive to shy, length of stay varied from seconds to hours.

Flowering sequence had a direct influence on the grouping of umbel visitors as potential pollinators or visitors. Only insects, visiting the umbels in both flowering stages and regularly touching the reproductive surfaces could be pollinators. Except one bee species (*Dufourea* sp., Fig. 12J), these insects belonged to beetle families (Tables 4 and 5). Six species were identified as most likely regular pollinators being frequently present and switching among umbels. A second, less effective group of nine species was not always present or stayed for a long time on the umbels. Insects that not always contacted the reproductive surfaces or visited only rarely the umbel in the receptive stage, might act as occasional pollinators. Visitors were insects and spiders rarely observed, only visiting pollen-presenting umbels or not contacting the reproductive surfaces.

##### Manipulation test:

Among the five beetle species assumed to be good pollinators, *Plagionotus floralis* (Fig. 12B) was the largest (up to 2 cm) and due to its bee-like pattern a very conspicuous one. The beetle moved slowly across the umbel, thereby getting loaded with pollen all around its body. He showed an aggressive behaviour chasing other umbel visitors away (Table 4). *Anthaxia* sp. (Fig. 12E), in contrast, was almost all the time fast moving across the umbel. It was rather shy flying away immediately when other visitors approached too closely. The other three beetle species, *Mylabris quadripunctata* (Fig. 12C), *Hoshihananomia* sp. (Fig. 12A) and *Podonta* sp. (Fig. 12F) did not show a noticeable behaviour. They were moving slowly across the umbel feeding from pollen or copulating with a mating partner.

We found no response to our removal treatment (Table 6). All five species behaved as on the control umbels. However, in the treatment with enlarged dark centres, two beetle species showed a clearly changed behaviour. *Plagionotus floralis* visited the manipulated umbel more often and attacked the brushes as it did with umbel visitors. In contrast, the shy *Anthaxia* sp. was found less frequently on these umbels.

**Table 6.** Results of the manipulation experiments. Average number of visits per pollinator species and umbel during 5 days, twice the day for 20 min, June 2014. Once landed on the umbel, the residence time of the beetle individuals varied considerably.

	Control	Brush removed	Brushes added
*Anthaxia* sp.	166	172	70
*Plagionotus floralis*	62	62	93
*Mylabris quadripunctata*	54	48	41
*Hoshihananomia* sp.	46	34	36
*Podonta* sp.	18	18	17

Being aware that this experiment had a preliminary character, we intended to repeat the treatments in 2015. However, we found the range of visitor species completely changed. *Plagionotus floralis* and other species observed in 2015 were lacking, whereas new beetle species from Curculionidae, Cerambycidae, Buprestidae and Scarabaeidae and more fly species were present. Without renewed data on the behaviour of the umbel visitors, we could not repeat the experiment.

#### Case study II.

*Echinophora trichophylla* is a perennial plant species endemic to Turkey. We only found one population close to Küplüköy village (358 m) in NW Anatolia where the plants grew on stony hills along the roadside (Fig. 5A).

**Figure 13.**
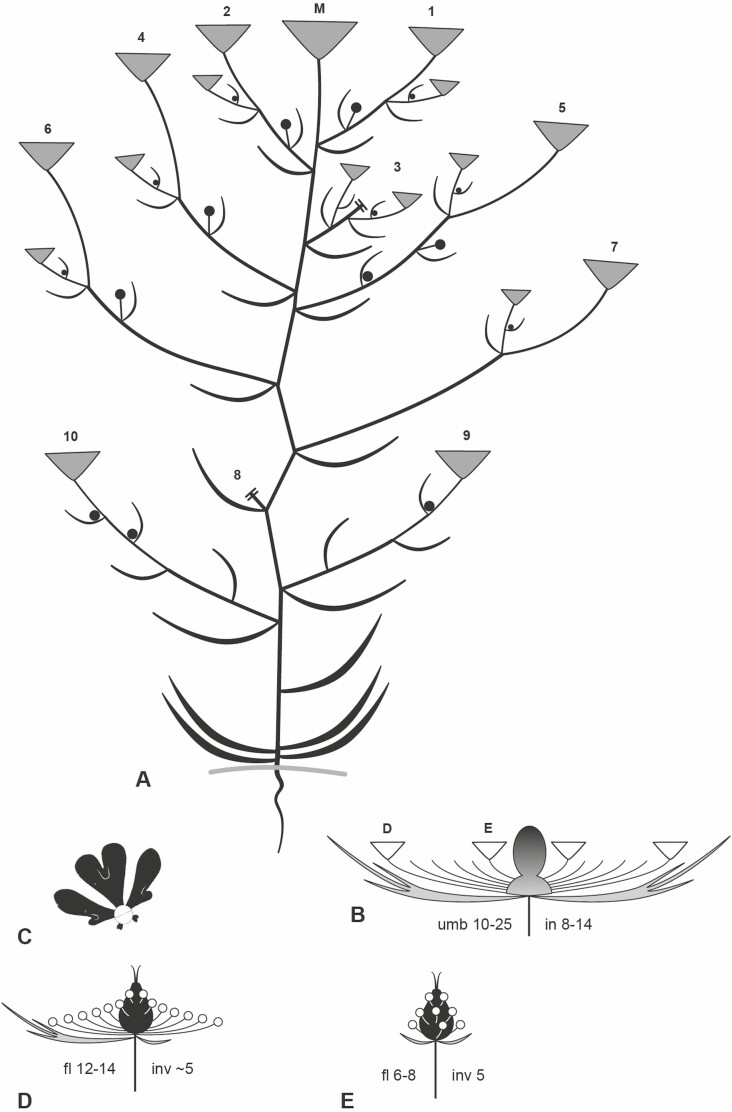
*Echinophora trichophylla*. Architecture and umbel construction. (A) Side-view of a moderately sized individual with a main umbel (M), 10 umbels of first branch order (1–10) and several umbels of second order. (B) Side-view of an umbel with the prominent plug in the centre. Only a few umbellets are delineated, D and E refer to details given in D and E. in, number of involucral bracts. umb, number of umbellets. (C) In each ray flower, one bilobed and two one-lobed petals are enlarged. (D) Characteristics of a peripheral umbellet with a large perfect flower in the centre and up to 14 staminate flowers. fl, number of flowers. inv, number of involucellar bracts. (E) Side-view of an inner umbellet with less staminate flowers.

In June 2015, *E. trichophylla* was the dominant species with a moderate population size of 20–30 individuals. The plants were well developed usually reaching a height of 1 m. Individual plants were highly branched (Figs 5B and 13A) presenting on average 50 umbels (*n* = 5). The main umbel was enriched by 8–12 lateral umbels of first, 20–40 of second and 0–8 of third branch order. In June 2016, only five large plants and a few smaller individuals (up to 40 cm) were present. In contrast, co-flowering individuals of *Daucus guttatus* (with white- and dark-centred umbels, respectively), *Artedia squamata*, and, in a small number, *Daucus carota* subsp. *carota* were much more frequent than in the year before.

##### Architecture, flowering sequence and fruit set.

Architecture, andromonoecy, protandry and ordinal flowering sequence largely resembled *Artedia squamata*. However, besides the different patterns of ray flower formation and the plug-like structure in the umbel centre (Fig. 13B and D), flower morph distribution was considerably distinct. In each umbellet, only the terminal flower was perfect whereas all other flowers, including the ray flowers, were functionally or completely staminate (Fig. 13D). Peripheral umbellets (up to 14 flowers) were larger than the innermost ones (6–8 flowers). The degree of andromonoecy, that is, the percentage of staminate to perfect flowers in an individual plant, was with 89 % (*n* = 3) higher than in *Artedia squamata*.

Flowering began in the main umbel with pollen presentation. The styles started to elongate at the end of this phase and the stigma became receptive when all pollen was taken away. Due to this phase separation and the ordinal flowering sequence, the plants exhibited multicyclic protandry.

The fruit set was high, almost 100 % in the first umbels and slightly less in the umbels of higher branch order (Fig. 15I). Usually, the central flower of each umbellet developed a fruit. As the inferior ovaries were congenitally sunken in the umbellet receptacles, the swelling of the maturing fruit elevated the persisting staminate flowers (Figs 13D and 15I). Their pedicels and involucellar bracts became woody and spiny protecting the developing fruit and contributing to its epizoochorous propagation.

##### Umbel visitors and potential pollinators.

**Figure 14.**
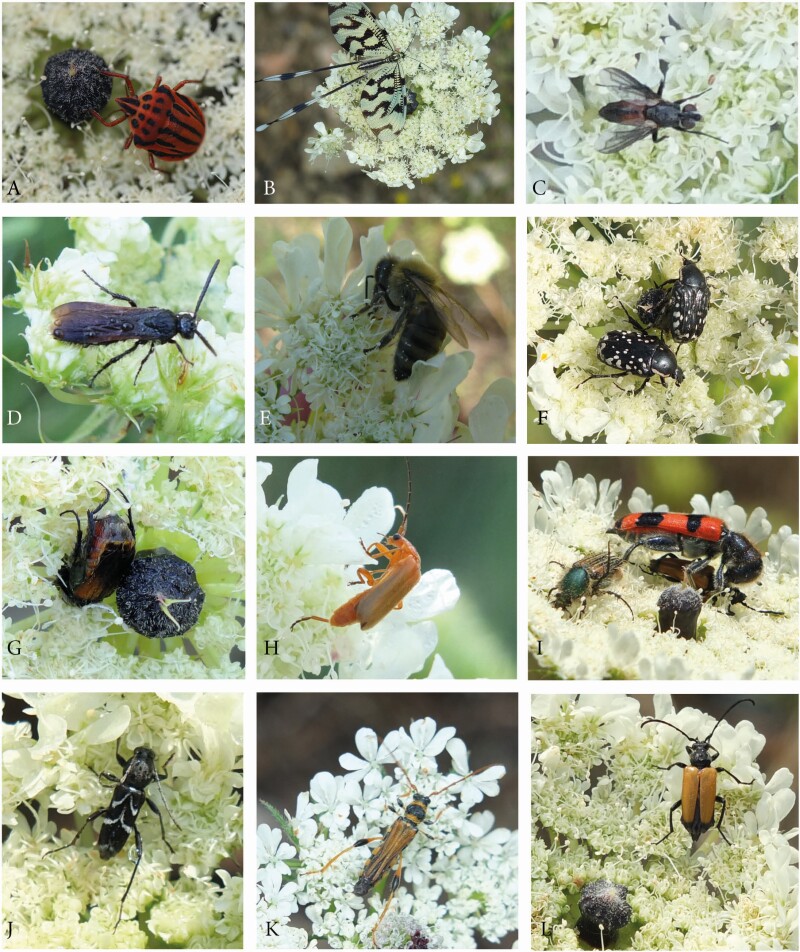
*Echinophora trichophylla.* Umbel visitors. (A) *Graphosoma lineatum,* Hemiptera. (B) *Nemoptera sinuata,* Neuroptera. (C) Fly. (D and E) Bees. (F–N) Beetles. (F and G) Scarabaeidae. (F) *Oxythyrea funesta.* (G) Scarab beetle hiding in the umbel. (H) *Rhagonycha fulva*, Cantharidae. (I) *Trichodes* sp., Cleridae, with prey. (J–L) Cerambycidae. (J) *Clytus* sp. (K) *Stenopterus* sp. and (L) *Paracarymbia fulva*.

**Figure 15.**
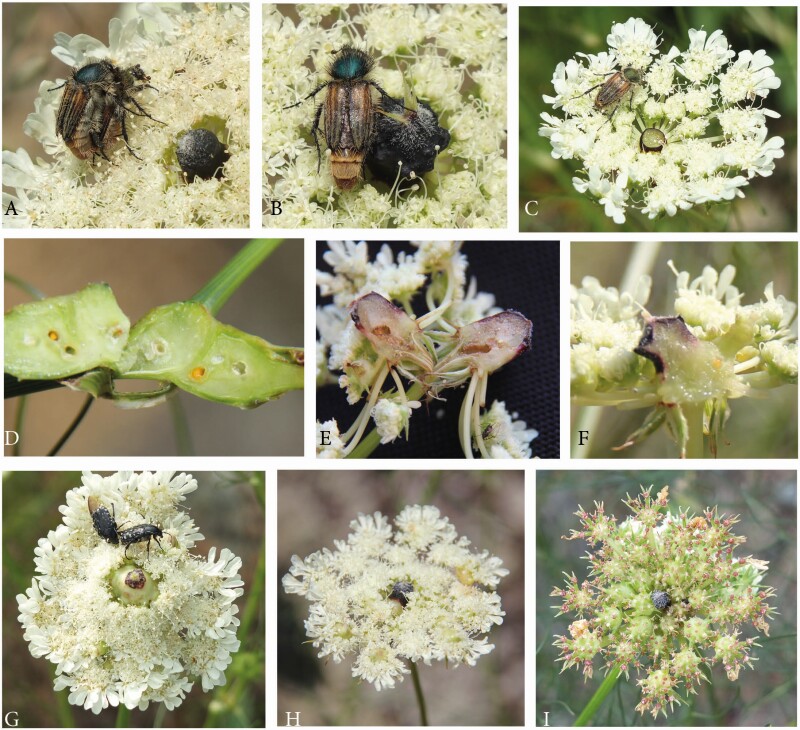
*Echinophora trichophylla*. (A–C) *Eulasia nitidicollis,* Glaphyridae. (A) Copulation on the umbel. (B) Male beetle attracted by the black plug. (C) Female beetle feeding on an umbel with removed plug. (D) Gall infection. Longitudinal section of a shoot base showing the swollen tissue. (E and F) Longitudinal section of central structures. (E) Gall. (F) Natural plug. (G and H), Gall infection. (G) Umbel with a gall in its centre. (H) Umbel with galls in the umbellet centres. (I) Not infected umbel in the fruiting stage; the umbellets are swollen due to the developing fruit in their centres.

Umbels were visited by many different insects. On the first view, diversity was similarly high as in *Artedia squamata*, but due to the short observation time of only a few days, we could not list all species. Bugs (Hemiptera: *Halyomorpha* sp., *Graphosoma lineatum*, Fig. 14A) and Neuroptera (*Nemoptera sinuata*, Fig. 14B) were insect groups not observed on *A. squamata*. Flies and bees were common, but beetles were by far the most dominant group (Fig. 14). They fed from pollen and nectar, waited for mating partners or hide within the umbel. *Trichodes* sp. (Cleridae), a rather large and conspicuous beetle, watched for prey. It bit the victims in the neck and then ingested the nutritive components. *Oxythyrea funesta* (Scarabaeidae, Fig. 14F) was common, often inspecting the dark plug before feeding or copulating. The most common visitor and most likely a good pollinator due to its hairy body and presence on pollen-presenting and receptive umbels was *Eulasia nitidicollis* (Glaphyridae, Fig. 15A–C).

In this beetle species, males and females are easily distinguishable. Females have a light brown elytron and a pale greenish pronotum, whereas males are a bit darker brown with a green-metallic pronotum (Fig. 15B and C). Females landed anywhere on the umbel and started feeding on pollen. Males, in contrast, were predominantly searching for females. They either landed on an umbel close to a female and immediately started to copulate (Fig. 15A) or, in case no female was around, close to the dark plug (Fig. 15B). After a short inspection, they fed on pollen.

This behaviour indicated that males were much more attracted by the dark plug than females. To test this observation, we spontaneously removed the dark plug from 20 umbels standing nearby and recorded the frequency of visits and the behaviour of the beetles of *E. nitidicollis* for 30 min. Results were then compared with data collected on 20 untreated umbels under the same conditions.

Females were not affected by the plug removal. They landed on the test umbels and behaved just as on untreated ones. In contrast, male beetles were never seen on a manipulated umbel. They only landed on such an umbel when a female was present as a mating partner. We preliminarily concluded that male beetles were attracted by the dark plug because they perhaps took them for females from a distance. We again intended to repeat the experiment in 2016, but as in *Artedia squamata*, the insect fauna was remarkably different among the seasons. There were clearly less insects on the umbels and, most important for our study, *Eulasia nitidicollis* was completely lacking.

##### Gall infection.

In 2016, the umbels of *E. trichophylla* (and no other plant species around) were heavily infected by galls. Vegetative parts like leaf bases, nodes or stem parts were swollen and showed traces of infection when longitudinally dissected (Fig. 15D). The umbels responded with white swellings of various size, shape and colour, which appeared at different positions depending on their age.

In young, still developing umbels, the umbel centre was infected. The receptacle bulged and formed a white swelling (Fig. 15G) or a dark plug. In this case, longitudinal sections were needed to confirm gall infection (Fig. 15E and F). In slightly older umbels, the centre of the umbellets was infected (Fig. 15H). As this was the place of the only perfect flower per umbellet, the infection had considerable effects on fruit set. In the extreme case, an infected umbel did not produce a single fruit. The relative timing between infection and umbel development was also obvious when comparing the umbels within a single individual. Due to modular architecture and ordinal development, the umbel orders differed in age. In a representative plant, we found no infection in the main umbel. The 11 first-order umbels were either not infected (*n* = 5) or had galls only in the umbellet centres (*n* = 6). The 28 umbels of the second order were rather diverse: 12 umbels were not infected, 4 showed a gall in the umbel centre, 6 in the centre of the umbellets and 6 in both the umbel and umbellet centres. The six umbels of third order, still developing, were all infected in the umbel centre. It was evident that gall infection started at the end of the first order umbel development and fully affected the second- and third-order umbels.

## DISCUSSION

The present paper illustrates that the wild carrot *Daucus carota* is neither the only species with dark-centred umbels nor a typical representative of this group. It has a terminal umbellet, coloured flowers and a labile formation of the dark centre, whereas the other species lack a terminal umbellet and have morphologically diverse dark centres in all umbels.

### Apioid species with dark-centred umbels

**Figure 16.**
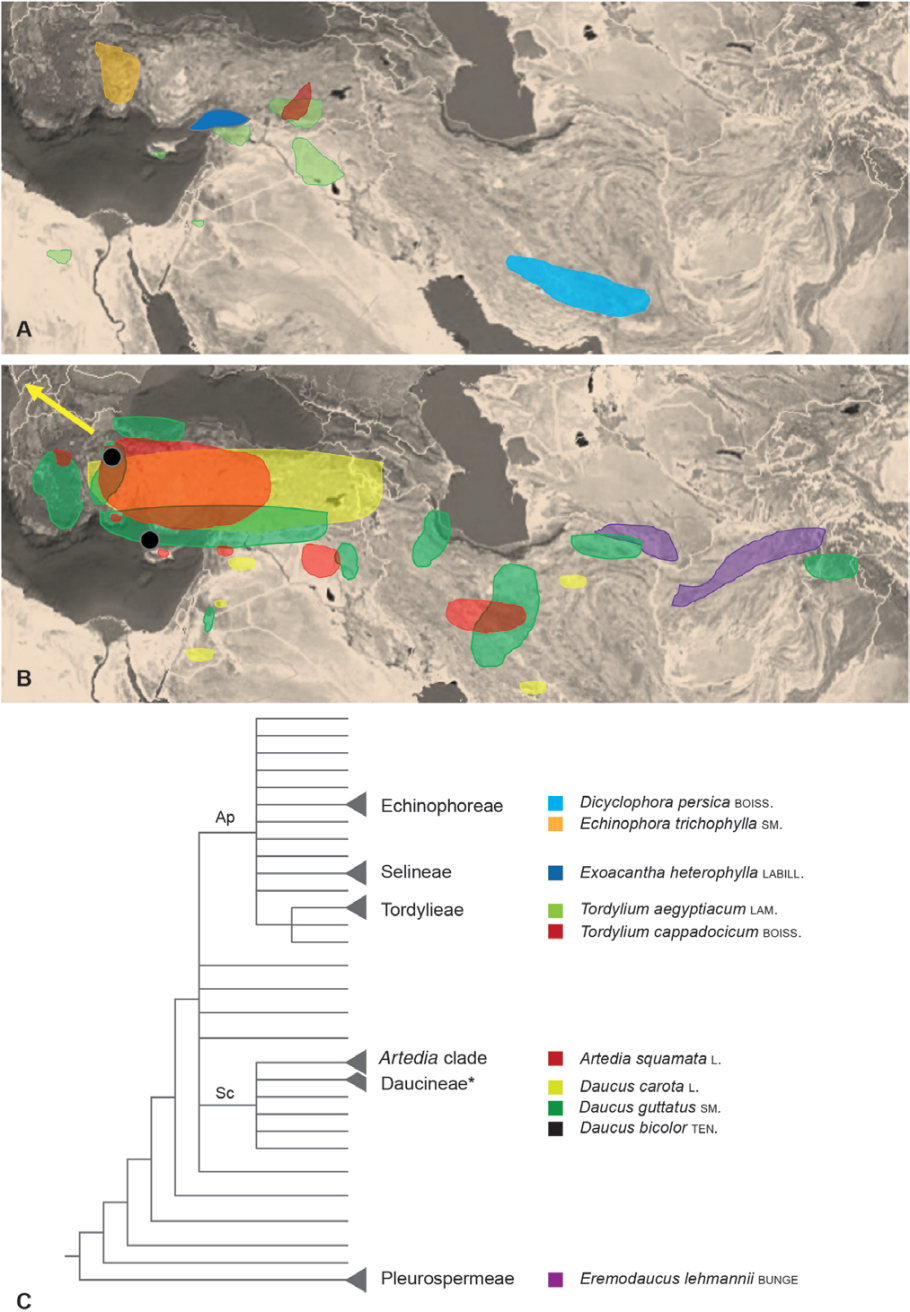
Distribution areas and phylogenetic relationship of dark-centred Apiaceae. (A and B) Except *Daucus carota* with a wide-spread distribution (arrow), all species are native to an area extending from the E Mediterranean to C Asia. The black spots in B indicate the localities of the populations investigated in *Daucus guttatus*. (C) Species belong to six different subclades, three nesting within the Apioid superclade (Ap), two in the Scandiceae (Sc) and one in the Pleurospermeae. *Further *Daucus* species mentioned in the discussion. Colours correspond to those used in A and B. Distribution based on local floras (see ‘Methods’ section). Map after Rechinger and Hedge (1987). Phylogeny after Downie *et al.* (2010).

In total, 10 species (one of them with two subspecies) from 7 genera and 6 clades could be identified as producing dark-centred umbels (Fig. 16C). Except *Eremodaucus lehmannii* and *Exoacantha heterophylla*, they were all found in the field. The reconstruction of their distribution area indicates that except the wild carrot all species are native to an area extending from E Mediterranean to C Asia. The centre of diversity is Anatolia (Fig. 16A and B). Except *Echinophora trichophylla*, all observed species are monocarpous, that is, annual or biannual. They share a modular architecture, andromonoecy, ordinal flowering sequence and multicyclic protandry, grow in large populations and depend on pollinators.

#### What is known about the species not investigated in the present study?

*Eremodaucus lehmannii* and *Exoacantha heterophylla* are annuals with flat, white (or sometimes reddish) umbels showing a red centre. Ray flowers are lacking.

**Figure 17.**
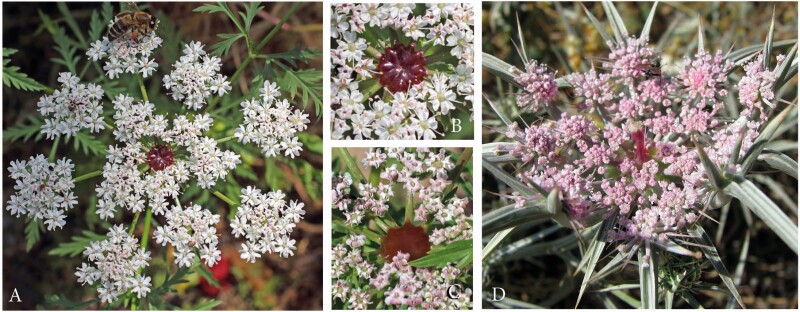
Species not investigated in the present study. (A–C) *Eremocaudus lehmannii.* (A) Umbel with red centre in top view. (B) Detail of (A) showing the swollen bases of the raylets. (C) Centre of another umbel with an unstructured red spot covering the whole area. (D) *Exoacantha heterophylla*. Umbel with a massive red structure in its centre. (A–C) By courtesy of Alim Gaziev, Taschkent (Uzbekistan). (D) By courtesy of Noam Avitsel, Pardes Hana (Israel).

*Eremodaucus lehmannii* is a weed from central Asia inhabiting semi-deserts, dry steppes, road edges and waste areas (Plunkett *et al.* 2018). Umbels show a reddish centre composed of swollen bulges arranged around the sterile centre (Fig. 17A and B). In contrast to all species investigated, these conspicuous structures are neither flowers nor central excrescences, but formed by the bases of the umbellet stalks (Troll and Heidenhain 1951). In some umbels, the bases merge into a large red, unstructured spot covering the whole umbel centre (Fig. 17C).

*Exoacantha heterophylla* is native from southern Turkey to Syria and grows as an erect, spiny weed along roadsides and pastures (Davis 1972). The umbel centre is dominated by a compact, red structure (Fig. 17D). Schmidt and Magin (1997) illustrate and describe the central structure based on herbarium material (Fig. 1B). The basal part is winged, the middle part covered by clubbed hairs or emergences and the tip of the structure bears some free or fused bristles. Altogether, the central structure resembles the one of *Echinophora trichophylla* indicating that *Exoacantha heterophylla* fits well with the other species of the Apioid superclade (Fig. 16C).

#### Phylogenetic position and peculiarities of the species.

The species with dark umbel centres belong to six different subclades of the Apiaceae-Apioideae and to seven genera nesting within the Apioid superclade, the Scandiceae and Pleurospermeae (Fig. 17C: Ap, Sc). All species of the Apioid superclade have compact structures, whereas the remaining ones are more diverse, presenting coloured flowers or umbellets (*Daucus*), brush-like structures (*Artedia*) or swollen raylet bases (*Eremodaucus*). Based on phylogeny and morphological diversity, it is evident that the dark umbel centres evolved several times in parallel.

Five of the seven genera with dark centres are either monotypic (*Dicyclophora, Exoacantha, Artedia, Eremodaucus*) or have only a single species with dark-centred umbels (*Echinophora*). Among the ca. 20 species of the genus *Tordylium*, only *T. aegyptiacum* was known to produce a dark structure in the umbel centre (Schmidt and Magin 1997). In the present study, we identified *T. cappadocicum* as a second example. As umbel colouring is often not included in taxonomic descriptions, it is likely that more species with dark-centred umbels may exist.

#### The Daucus alliance.

The genus *Daucus* includes several species with dark flowers. Schmidt and Magin (1997) listed *D. carota*, *D. guttatus* and *D. broteri* as examples, Martinez-Flores *et al.* (2016) added *D. bicolor*, *D. conchitae* (Turkey and Aegean Islands), *D. gracilis* (Algeria) and *D. setulosus* (E Mediterraneum). However, looking at the taxonomic literature, it is difficult to visualize the umbels of these species. In the classical floras, fruit spines and involucre morphology are generally used for discriminating *Daucus* species, whereas umbel characteristics are largely disregarded. Furthermore, some species identified in molecular phylogenetic studies lack morphological diagnostic characters. For example, the complex of *Daucus guttatus*, *D. bicolor* and *D. broteri* has been discussed repeatedly (Arbizu *et al.* 2016; Martínez-Flores *et al.* 2016). Whereas Arbizu *et al.* (2016) included *D. broteri* into *D. guttatus*, Banasiak *et al.* (2016) and Martinez-Flores *et al.* (2019) kept both species. The main reason is that *D. bicolor* and *D. guttatus* are phylogenetically close together and distributed in E Mediterraneum whereas *D. broteri* is a bit more distant and restricted to Italy.

In some species, dark-centred umbels appear to be only attractive variants of otherwise white umbels. Martinez-Flores *et al.* (2016) mentioned that *D. broteri* has usually white umbels, but that some plants produce umbels with one or few coloured flowers in the centre. They continued that *D. bicolor* usually shows a large, dark centre which, however, is lacking in some individuals. Reduron (2007), in contrast, mentions that its flowers are usually white and only sometimes pink. We found such variable colouring in *D. guttatus*. Populations either include only dark-centred individuals (as found in Side) or are mixed (as in Küplüköy) or completely white flowering (as concluded from Davis (1972), not mentioning dark centres for this species). Obviously, there is a high degree of variation that might be due to internal and/or external conditions. It is not known so far what stimulates the colouring of the umbels.

The dark colour comes from anthocyanidin (Troll and Heidenhain 1951), which is also found in the roots of carrot (Harborne 1976) and, occasionally, in reddish-toned umbels. Anthocyanidin has an antioxidant effect and may be produced in these umbels as a response to physiological stress (Buchanan *et al.* 2000). Hinderer *et al.* (1983) showed that white and red petals in *D. carota* only differed in the activity of the main enzyme triggering pigment synthesis. Variation in enzyme activity may, thus, explain the facultative formation of red umbel centres.

The presence versus absence of a dark flower in the umbel centre of *D. carota* is known for centuries. Dioskorides and Albertus Magnus already observed the dark flower (cited after Troll 1957) that was repeatedly mentioned from the 17th century onwards (Reduron 2007). The number of dark flowers per umbel varies from one to few (rarely increasing up to 17, Daumann 1973), the percentage of dark-centred umbels in a population from less than 20 % to over 90 % (Germain de Saint-Pierre 1854; Schulz 1888; Detto 1905; Troll 1957; Néhou 1961; Daumann 1973; Eisikowitch 1980; Westmoreland and Muntan 1996). Our study resulting in 72 % of individuals with dark flowers additionally considered the position of the dark-centred umbels within and among the plants. The finding that red flower formation depends on umbel size and plant vigour is in accordance with the suggestions of Gonzalez *et al.* (2018) that plant developmental factors may affect the expression or suppression of red flowers.

Within the genus *Daucus*, the pattern changes from single red flowers in terminal position to several coloured umbellets arranged laterally in the umbel centre. Interestingly, Banasiak *et al.* (2016) recognizing 26 species in their molecular analysis, found *Daucus* species falling into different sections. *Daucus carota* with red terminal flowers was placed in section *Daucus* (together with *D. gracilis* and *D. setulosus*), whereas *D. bicolor* and *D. guttatus* with colored umbellets were placed in sect. *Anisactis* (together with D. *broteri* and *D. conchitae*). This finding looks like a phylogenetic signal, but needs confirmation by future studies.

### Homology of dark umbel centres

The apioid umbel is unique among angiosperm inflorescences in producing massive dark structures in its centre. Whereas, in the past, most attention has been given to the wild carrot, the present study illustrates that diversity of dark structures is much higher than expected. It falls into four groups. The *Daucus* group is characterized by dark flowers and umbellets, *Eremodaucus lehmannii* by coloured raylet bases, *A. squamata* by a blueish brush and the remaining species by massive structures of various shapes. It is evident that the dark structures evolved in parallel, but almost nothing is known about their development and morphological homology.

#### Umbel development from flower-like meristems.

Traditionally, the umbel with umbellets has been interpreted as a branched inflorescence derived from a panicle or thyrse (Froebe 1971). However, recent ontogenetic investigations show that the umbel does not develop from an inflorescence meristem, but rather from a flower-like ‘floral unit meristem’ (Claßen-Bockhoff and Bull-Hereñu 2013; Claßen-Bockhoff and Arndt 2018). Our studies in *Artedia squamata* and *Echinophora trichphylla* confirm the finding of Baczyński *et al.* (2022) that umbellets and flowers are not segregated from an acropetally developing inflorescence meristem, but originate from a determinate umbel meristem by the process of fractionation (Claßen-Bockhoff and Frankenhäuser 2020). The meristem expands and splits into submeristems (umbellet meristems), which enlarge and split into flower meristems, which, finally, after further meristem widening, initiate floral organ primordia. Umbel development is, thus, characterized by repeated steps of meristem expansion and fractionation, before floral organs are formed; it shares these processes with flower development and only differs from it in the number of fractionation steps (Claßen-Bockhoff 2016).

Floral unit meristems lack apical growth but expand during development. The newly generated space stimulates fractionation which is an auxin-dependent, self-autonomous process (Reinhardt *et al.* 2003: Runions *et al.* 2014) responding to the geometrical conditions of the meristem (Prusinkiewicz and Barbier de Reuille 2010). This has been quantitatively shown in *Daucus carota* by comparing the development of open (no terminal flower) versus closed umbellets (with terminal flowers; Bull-Hereñu and Claßen-Bockhoff 2010). Open umbellet meristems are small and flat until they are completely used by flower meristems. There is no space left in the centre to form a terminal flower. In contrast, the centre of the larger closed umbellet meristems becomes dome-shaped and large enough to form the terminal flower. The same difference in meristem shape was found in other apioid species with always open versus closed umbellets pointing to the general importance of meristem geometry (Bull-Hereñu and Claßen-Bockhoff 2010). Transferred to the umbel meristem, we conclude that the facultative formation of terminal flowers in *D. carota* may depend on meristem size and available space. This not only corresponds to our macromorphological observations but also indicates that umbel size does not matter in terms of flower number, but of available space left after the last lateral umbellet initiations.

#### Changes in umbel development promote receptacle excrescences.

 Our developmental studies in *Artedia squamata* and *Echinophora trichophylla* do not fit the general umbel development described above. The umbel meristem ceases umbellet fractionation before the meristem has been completely used leaving a large naked centre. In *Artedia squamata*, the available space is used by random primordia fractionation resulting in the brush which is later elevated by receptacular growth. In *Echinophora trichophylla*, the naked centre bulges earlier only occasionally fractionating bract-like structures. Random primordia fractionation and meristem elongation without further primordia formation resemble mutants described from *Gerbera* (Asteraceae; Zhao *et al.* 2016) and *Arabidopsis* (Brassicaceae; Reinhardt *et al.* 2003). Though we do not know the genetic regulation of the dark centres in apioid umbels, we assume that the unique structures are the evolutionary outcome of a combination of specific floral unit meristem conditions, mutations and autonomous growth processes using available space.

Given that the umbel development of *Artedia squamata* and *Echinophors trichophylla* represents that of *Tordylium, Exoacantha* and *Dicyclophora* species, we summarize that the formation of dark umbel structures appears to be linked with spatial conditions at the umbel meristem. The massive outgrowths are clear examples of parallel evolution, that is, the independent formation of morphologically homologous structures. However, compared to the coloured flowers in *Daucus* and the swollen raylet bases *in Eremodaucus*, the brush and plugs have nothing in common with the development of an umbellet. They offer a nice example of convergent evolution, that is, the formation of functionally similar but analogous structures.

### Adaptive value of the dark umbel centres

The parallel and convergent evolution of dark structures in several lineages of the Apioids raises questions. Given that these structures evolved randomly by mutations, it is likely that their distinct form and presence in all umbels and individuals of a population may be maintained and stabilized by natural selection. What is the adaptive value of the dark umbel centres and why are they restricted to a relatively small geographic area?

#### Dark-centred umbels are adaptive but not addressed to a single pollinator guild.

 Whereas some authors concluded that the dark flowers in *Daucus carota* might have no adaptive significance for pollination and seed set (Darwin 1877; Daumann 1973; Polte and Reinhold 2013), other researchers believed that they were adaptive. They tested the *flycatcher effect* (Eisikowitch 1980) if the dark centre would mimic conspecifics and release aggregative behaviour of pollinators. They observed insect numbers and behaviour on natural white- versus dark-centred umbels, removed red flowers and added dead insects or coloured papers to test the long-distance attraction of the dark centre to umbel visitors (Westmoreland and Muntan 1996; Lamborn and Ollerton 2000; Goulson *et al.* 2009; Gonzalez *et al.* 2018). Their results were disillusioning. In each study, some insect species were found to be affected by the dark centre, whereas the majority were not. Moreover, umbel visitors differed among localities (Westmoreland and Muntan 1996) and seasons (Lamborn and Ollerton 2000). In Portugal, the beetle *Anthrenus verbasci* (Dermestidae) was by far the most frequent umbel visitor in the first season and completely lacking in the following year (Goulson *et al.* 2009).

Our study largely confirms the results of previous studies and adds further observations. We found many highly diverse visitors on the umbels of all species observed. In *Artedia squamata* and *Echinophora trichophylla,* species composition varied considerably among seasons. In both species, the dominant visitors of the first season, *Plagionotus floralis* (Cerambycidae) and *Eulasia nitidicollis* (Glaphyridae), were not present on the umbels in the second year of observation. Furthermore, we found proofs for long-distance effects in *Daucus carota* subsp. *carota* attracting male *Hylaeus* bees in NW Turkey and in *Echinophorea trichophylla* attracting male individuals of *Eulasia nitidicollis* beetles. The behaviour of the male insects including the direct approach to the dark structures and their careful inspection indicated that they were most likely searching for a mating partner. However, pseudocopulation as described in orchids (Schiestl 2005) and *Gorteria diuffusa* (Asteraceae, Ellis and Johnson 2010) has never been observed in Apiaceae.

Apart from long-distance attraction of potential pollinators, short-distance effects have been discussed probably influencing the behaviour of umbel visitors (Leppik 1972) or biotic interactions among prey and predator species (Lamborn and Ollerton 2000). Polte and Reinhold (2013) recorded the location and orientation of landing and the visit duration of insects on main umbels with and without dark flowers; they found no evidence for the role of the dark flowers. However, our preliminary experiments in *Artedia squamata* indicated that the dark brush might influence the biotic interactions among umbel visitors. If the brush increases the switch-over of some visitors to umbels of neighboured plants, pollen dispension and, consequently, male fitness will be increased (Westmoreland and Muntan 1996).

Considering all species with dark-centred umbels, we found some similar characters including three-dimensional shape, dark anthocyanidin pigmentation, hairiness and the presence of stomata. Whereas the first characters may mimic a hairy conspecific or predator specimen, the role of the stomata is unclear. We speculate that they may cause a cooling effect due to transpiration (Azad *et al.* 2007), thereby signalling a sleeping place for some visitors, but it is also possible that they have no specific function (Hew *et al.* 1980).

Polte and Reinhold (2013) found that umbels of the wild carrot growing on the campus of Bielefeld University, Germany, were significantly less infected by the gall midge *Kiefferia pericarpiicola* than umbels without a dark centre. The galls are thick globular structures of red to violet colour resembling dark flowers. The authors concluded that one of the possible functions of the dark flowers might be mimicking a gall, thus reducing oviposition by gall midges. Indeed, as floral evolution is influenced by interactions with herbivores and pests (Galen 1999), protection against gall infection might be a further function of the dark flowers. We found a massive gall infection in *Echinophora trichophylla*, which was most likely caused by *Lasioptera* species often associated with Apiaceae in Turkey (Skuhravá *et al.* 2005). The larvae developed in both vegetative plant parts and umbels. The different responses of the *E. trichophylla* umbels to the infection indicated that the gall midges preferably laid their eggs in young developing tissue that was the umbel centre in a young umbel and the umbellet centre in an older one. In the second case, fruit set was severely affected as the perfect flowers were disturbed. However, given the perennial life form of the species, the population will survive if the infection by gall midges happens only occasionally.

Summarizing the data at hand, the general conclusion is that the dark centre is adaptive. It increases the general attractiveness of the umbels for diverse insects and may have specific functions for some of them. Though it affects not all visitors, it most likely enhances pollination under certain conditions (Goulson *et al.* 2009).

#### The number of visitors does not reflect the number of pollinators.

Lamborn and Ollerton (2000) determined the pollen load of the most abundant insects visiting the carrot umbel in England and found that the insects with the highest pollen load were not the most frequent ones. This observation corresponds to the findings of Bell and Lindsey (1978) studying American apioid species. The authors counted the pollen load of 55 species visiting the umbels of *Thaspium barbinode* and of 26 species observed on *Zizia trifoliata*. Though over 50 % of the visitors in both species were beetles, these insects only carried 2 % and 1 % of pollen. Over 85 % of pollen was loaded by hymenoptera that accounted for 40 % and 9 % of the visitors. Bell and Lindsey (1978) found similar examples in other apioid species and concluded that many Apiaceae may not fit the generalized pattern of promiscuous pollination.

Since at least Bell (1971) stated that each insect visiting an umbel might be a potential pollinator, apioid umbels were taken as examples for generalized pollination systems. In fact, none of the studies conducted on *Daucus carota* distinguished between visitors and pollinators. They disregarded the fact that in extremely protandrous species like *Daucus carota* (Koul *et al.* 1989), only those insects can be pollinators which visit the umbel in both flowering phases, that is, in the pollen-donating and the pollen receptive stage (Zych 2007; Niemirski and Zych 2011).

Our analysis in *Artedia squamata* elucidated that the flowering sequence resulted in temporal dioecism (Cruden and Hermann-Parker 1977). Among the more than 50 visiting species only 6, i,e., 5 beetle and 1 bee species, were regularly seen on both umbel stages transferring pollen. As they were clearly loaded with pollen and touched the receptive surface of the stigmas, we concluded that they were potential pollinators. Further 15 beetle species were either powdered with less pollen or appeared more rarely on umbels in the receptive stage. They were assumed to be occasional pollinators, whereas all other visitors were either only present on the pollen-donating umbel or not able to transfer pollen due to behaviour and/or body proportions. It is, thus, evident that the sheer number of umbel visitors does not reflect the number of potential pollinators.

Given the high fluctuation in the presence and abundance of insects among localities and seasons, it is also evident that the plants have not closely adapted to single pollinator species. They, instead, attract as many insects as possible. Even if most of them are pollen and nectar thieves, the unspecific attraction increases the chance that pollinators are among the visitors. Successful pollen transfer is particularly important for the apioid species with dark-centred umbels as they are all monocarpous (except *Echinophora trichophylla*) requiring a high level of seed set in each season.

#### Dark-centred umbels may function as beetle marks.

One of the most interesting findings of our study is the geographical restriction of the apiod species with dark-centred umbels to an area extending from the E Mediterranean to central Asia. The only exception is *Daucus carota* (native to Asia, Europe and North Africa, and introduced to North America, Australia, New Zealand and South Africa. Gonzalez *et al.* 2018). The distribution area is linked to the observation that beetles were by far the most abundant visitor species. Beetles belonged to more than 10 families, among them the Glaphyrinidae and Scarabaeidae whose hairy representatives are well-known pollinators (Kugler 1970; Dafni *et al.* 1990).

In the past, beetles were taken as occasional visitors only rarely contributing to pollination (Kugler 1970). However, Dafni *et al.* (1990) illustrated that red, bowl-shaped flowers in the E Mediterranean area were primarily pollinated by beetles (Glaphyridae) and only secondarily by bees. Most of these beetle-pollinated flowers are scentless, have a dark centre and lack UV reflectance. The example of *Papaver rhoeas* illustrates that these characters might be adaptive. Its flowers have black ‘beetle marks’ in the E Mediterranean area, but largely lack them in Europe. The loss goes along with UV reflection and bee pollination. Bowl-shaped beetle-pollinated flowers were called *painted bowls* by Bernhardt (2000). They evolved many times in parallel in the E Mediterranean area and in S-Africa (Dafni *et al.* 1990; Steiner 1998; van Kleunen *et al.* 2007). They attract beetles from different families. Only recently, Martinez-Harms *et al.* (2012) detected trichromatic vision in *Pygopleurus israelitus* (Glaphyridae) with the highest sensitivity in the UV, green and red area of the spectrum. Johnson and Midgley (1997) and van Kleunen *et al.* (2007) confirmed by experiment that monkey beetles (Scarabaeidae, Hopliini) were attracted by dark spots.

The appearance of apioid species with dark-centred umbels in an area known as a hotspot of beetle pollination (Bernhardt 2000) gives rise to the suggestion that the dark umbel centres might act as beetle marks. Except the red flower colour, the umbels share the lack of scent (Lamborn and Ollerton 2000) and UV reflection (Bell and Lindsey 1978) with the painted bowls and the dominance of beetle visitors. Moreover, populations of the wild carrot occurring outside the Mediterranean area, lack the dark centre in part of their individuals and are mainly visited by short-tongued bees and flies (Daumann 1973; Lamborn and Ollerton 2000; Ahmad and Aslam 2002; Pérez-Bañón *et al.* 2007) and less often by beetles (Goulson *et al.* 2009). We assume that the dark-centred umbels were stabilized by selection in the area of beetle pollination to increase the visitor rate.

## CONCLUSION

Putting together the data at hand, we conclude that dark structures in umbel centres evolved several times in different lineages of the Apioideae. They are morphologically diverse and in part based on mutative changes of floral unit meristem conditions. Given that they generally increase the attractiveness of umbels in areas with predominantly beetle pollinators, they may be stabilized by natural selection from E Mediterranean to Central Asian (Goulson *et al.* 2009). The dark centres are adaptive and can be functionally compared with beetle marks though they do not exclusively address beetles. The high number of attracted visitors is needed as most species are monocarpic and depend on regular seed set. Adaptive traits being addressed to a single pollinator guild should not be expected in such generalized systems (Ollerton 1996). Hence, the search for a single function of the dark-centred umbels was misleading from the beginning. The dark umbel centres serve more than one function and address diverse visitors depending on local and seasonal conditions.

## Data Availability

Plant vouchers are deposited at the Herbaria of Mainz University (MGU), Germany, Tehran (TARI), Iran and Kırıkkale University, Turkey. SEM probes are preserved as part of the Botanical Collections of the Johannes Gutenberg-University, Mainz. https://www.ub.uni-mainz.de/de/samml-ungen/botanische-sammlungen/4380.
